# Distinct immunological and molecular signatures underpinning influenza vaccine responsiveness in the elderly

**DOI:** 10.1038/s41467-022-34487-z

**Published:** 2022-11-12

**Authors:** Peggy Riese, Stephanie Trittel, Manas K. Akmatov, Marcus May, Jana Prokein, Thomas Illig, Christoph Schindler, Birgit Sawitzki, Yassin Elfaki, Stefan Floess, Jochen Huehn, Adrian J. Błażejewski, Till Strowig, Esteban A. Hernandez-Vargas, Robert Geffers, Bowen Zhang, Yang Li, Frank Pessler, Carlos A. Guzmán

**Affiliations:** 1 Department Vaccinology and Applied Microbiology, Helmholtz Centre for Infection Research (HZI), Inhoffenstraße 7, 38124 Braunschweig, Germany; 2 Research Group Biomarkers for Infectious Diseases, TWINCORE Centre for Experimental and Clinical Infection Medicine, Feodor-Lynen-Straße 7, 30625 Hannover, Germany; 3 Department Epidemiology and Health Care Atlas, Central Research Institute of Ambulatory Health Care, Salzufer 8, 10587 Berlin, Germany; 4 Center for Clinical Trials (ZKS), Early Clinical Trial Unit, Hannover Medical School, Feodor-Lynen-Straße 15, 30625 Hannover, Germany; 5 Hannover Unified Biobank, Hannover Medical School, Feodor-Lynen-Straße 15, 30625 Hannover, Germany; 6 Institute of Medical Immunology, Charité-Universitätsmedizin, Campus Virchow-Klinikum, Augustenburger Pl. 1, 13353 Berlin, Germany; 7 Berlin Institute of Health (BIH), Charité - Universitätsmedizin Berlin, Anna-Louisa-Karsch-Straße 2, 10178 Berlin, Germany; 8 Department Experimental Immunology, Helmholtz Centre for Infection Research (HZI), Inhoffenstraße 7, 38124 Braunschweig, Germany; 9 Cluster of Excellence RESIST (EXC 2155), Hannover Medical School, Carl-Neuberg-Str. 1, 30625 Hannover, Germany; 10 Department Microbial Immune Regulation, Helmholtz Centre for Infection Research (HZI), Inhoffenstraße 7, 38124 Braunschweig, Germany; 11 Centre for Individualised Infection Medicine (CiiM), Feodor-Lynen-Straße 7, 30625 Hannover, Germany; 12 Department of Mathematics and Statistical Science, University of Idaho, Moscow, ID 83844–1103 USA; 13 RG Genome Analytics, Helmholtz Centre for Infection Research (HZI), Inhoffenstraße 7, 38124 Braunschweig, Germany; 14 Department of Computational Biology for Individualised Medicine, Centre for Individualised Infection Medicine (CiiM), Feodor-Lynen-Straße 7, 30625 Hannover, Germany; 15 TWINCORE Centre for Experimental and Clinical Infection Research, Feodor-Lynen-Straße 7, 30625 Hannover, Germany; 16 Department of Internal Medicine and Radboud Center for Infectious Diseases, Radboud University Medical Center, Geert Grooteplein Zuid 10, 6525 GA Nijmegen, the Netherlands

**Keywords:** Vaccines, Adaptive immunity, Influenza virus, Computational models

## Abstract

Seasonal influenza outbreaks, especially in high-risk groups such as the elderly, represent an important public health problem. Prevailing inadequate efficacy of seasonal vaccines is a crucial bottleneck. Understanding the immunological and molecular mechanisms underpinning differential influenza vaccine responsiveness is essential to improve vaccination strategies. Here we show comprehensive characterization of the immune response of randomly selected elderly participants (≥ 65 years), immunized with the adjuvanted influenza vaccine Fluad. In-depth analyses by serology, multi-parametric flow cytometry, multiplex and transcriptome analysis, coupled to bioinformatics and mathematical modelling, reveal distinguishing immunological and molecular features between responders and non-responders defined by vaccine-induced seroconversion. Non-responders are specifically characterized by multiple suppressive immune mechanisms. The generated comprehensive high dimensional dataset enables the identification of putative mechanisms and nodes responsible for vaccine non-responsiveness independently of confounding age-related effects, with the potential to facilitate development of tailored vaccination strategies for the elderly.

## Abstract (web summary)

Seasonal influenza vaccination is an important strategy to prevent serious disease in the elderly, but individual responsiveness to vaccination widely vary. Here authors establish, with an array of state-of-the art methods, the major immunological parameters that distinguish vaccine recipients developing robust antibody response and non-responders

## Introduction

Seasonal influenza infections represent a global health threat with up to 3–5 million cases of severe illness annually leading to a large death toll and economic burden^1–4^. The elderly are one of the population groups at particularly high risk to develop severe disease, resulting in a huge number of hospitalizations and deaths. Currently, vaccination represents the most effective tool to contain seasonal outbreaks, which is especially recommended for risk groups. Unfortunately, vaccine responsiveness amongst the elderly is lower than in younger adults^5,6^. A population-based study in Scotland comparing individuals aged <65 with individuals aged >65 years revealed vaccine effectiveness against PCR-confirmed influenza cases of 60% versus only 19%, respectively^7^. Consequently, vaccines tailored to the specific needs of the elderly were developed to overcome the age-dependent reduced immune function. Fluad is an inactivated subunit vaccine adjuvanted with MF59C.1, an oil-in-water emulsion of squalene. It was shown to be superior to non-adjuvanted and high-dose antigen vaccines^8–11^. Studies aiming at elucidating the mechanisms underlying influenza vaccine responsiveness have usually compared immune responses in adults and elderly individuals, thereby primarily highlighting the setbacks of the aged immune system and completely neglecting to address effects within one age group^12,13^. This bias represents an important weakness, since such studies are not designed to elucidate immune mechanisms discerning between both elderly vaccine responders and non-responders. Thus, they are designed mainly to unravel age-related immunological deficiencies rather than other disguised mechanisms. Moreover, clinical trials investigating new or improved influenza vaccine formulations or vaccination strategies commonly focus on humoral immune responses as primary or secondary endpoints, to fulfill approval requirements. In-depth studies aiming at understanding the complex immunological mechanisms in their entirety are often missing. To overcome this bottleneck, in-depth immune profiling of human blood-derived lymphocytes, focused on addressing immunological and molecular aspects crucial for the initiation of protective adaptive immunity, is an emerging approach to evaluate the quality of vaccine-induced immune responses^14^. Systems vaccinology approaches, based on gene expression data, were used to identify predictive signatures for responsiveness to influenza or yellow fever vaccines, thereby demonstrating the usefulness of gene expression profiles as predictors for the response to vaccination^15–18^. In this context, a gene set enrichment-based approach proved valuable for overcoming the problem that subunit vaccines induce only small changes in gene expression profiles as well as the statistics-based selection of predictive genes not considering their biological impact^19^. This procedure led to the identification of gene signatures related to the proliferation of B cells that could predict antibody responses to influenza vaccination. Studies on adult influenza vaccinees further showed early signatures predicting later antibody titers as well as vaccine-induced signatures that were valid for various seasons and for diverse populations, including adults, elderly and diabetic individuals^20,21^. However, despite these crucial incremental insights, studies assessing differential vaccine responses or identifying predictive signatures for vaccine responsiveness within the group of the elderly are still largely missing. Such data, explaining inadequate immunity besides age-related factors, are urgently needed to improve vaccine efficacy in the elderly, since immune senescence does not necessarily explain the differences between aged vaccine responders and non-responders. Immune senescence is characterized by phenotypic and functional age-dependent alterations of different immune cell populations^22,23^. In addition to age-related immune senescence, individual cells can become exhausted, e.g., due to chronic stimulation. Replicative senescence, as characterized by loss of CD28 expression and expression of CD57, and functional exhaustion, marked by low responsiveness, are two manifestations of cell exhaustion that might also skew vaccine responsiveness^24–26^.

Besides the “classical” B and T cell populations, additional subsets are known to shape adaptive immune responses. T follicular helper (T_FH_) cells are essential for the initiation of B cell responses and thus represent another potential target for innovative vaccination strategies aimed at boosting humoral immunity^27,28^. The contribution of antigen-specific T_FH_ cells was shown to be important for mucosal antibody responses to a live attenuated influenza vaccine. Furthermore, individuals harboring higher numbers of dysfunctional T_FH_ cells show weaker vaccine responsiveness^29,30^. Besides the importance of functional immune activation, regulatory cells are crucial for the maintenance of balanced immune responses^31–33^. With regard to human circulating regulatory T (T_REG_) cells, no significant age-related changes in the frequencies were observed, and findings on functional properties are conflicting^34^. However, in the field of HIV vaccines, a correlation of T_REG_ cells and vaccination efficacy is evident and targeting T_REG_ cells is also moving into focus for the development of improved vaccines against other infectious diseases^35–37^. Regulatory B (B_REG_) cells were also shown to positively correlate with viral load, as well as to play a role in suppression of B and T cell responses in HIV and HBV patients^38–41^. However, in an HBV vaccination study, no correlation between IL-10-secreting B_REG_ cells and the serological response to the vaccine was found, thus their potential role during influenza infection as well as after vaccination remains elusive^42^.

The constantly evolving areas of multi-omics and mathematical modeling further boost research aiming at optimizing vaccination strategies by exploiting datasets from vaccinees^20,21,43,44^. Multi-omics-based approaches allow a comprehensive analysis considering various crucial aspects of the immune system. Our work is aimed to apply such multi-omics approach to influenza-specific vaccine responsiveness in the elderly, upon vaccination with Fluad^45^.

Here we present a biphasic prospective population-based study dissecting molecular and immune cell signatures of immune cell subpopulations by multi-parametric flow cytometry and multi-omics, including single-cell RNA sequencing and bulk transcriptome analysis, coupled to mathematical modeling. The study focus is the identification of critical parameters beyond immune aging to enable an assessment of the immune network in elderly individuals who are either vaccine responders or non-responders. The results show that in contrast to what was expected, poor vaccine responsiveness is not characterized by common age-dependent effects but is rather dictated by suppressive immune mechanisms, as confirmed by early immunological and genomic indicators. The identification of these mechanistic differences is instrumental for the development of tailored subgroup-stratified strategies to overcome poor vaccine-induced immunity.

## Results

### Identification and subgrouping of vaccinees into responders and non-responders

**Fig. 1:**
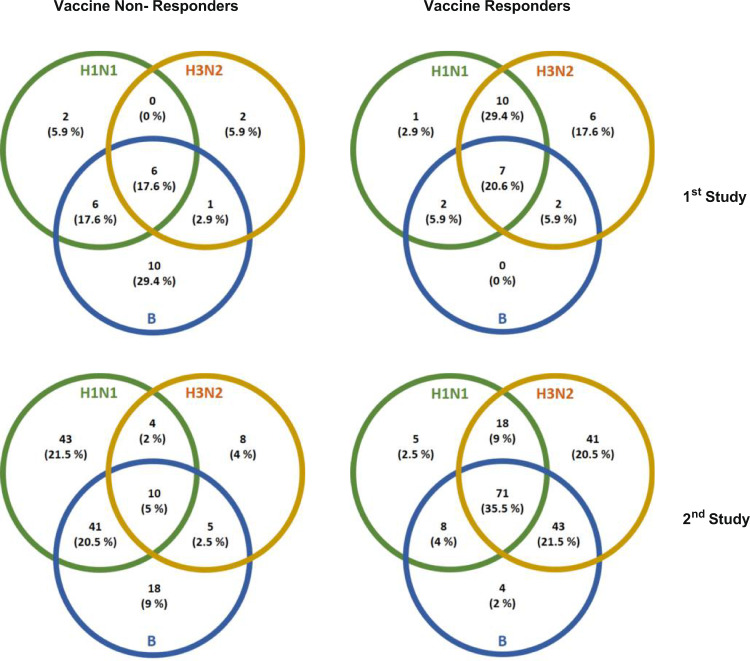
Stratification of vaccinees. Serum samples derived from vaccinees at baseline and 21 or 70 days after vaccination in the seasons 2014/15 and 2015/16 were used for quantification of influenza antigen-specific antibodies (HAI assay). Venn diagrams depict overlapping responses against the three vaccine antigens (1st study *n* = 34, 2nd study *n* = 200 biologically independent samples). Adapted from ref. 46, copyright by the co-author F.P. Source data are provided as a Source Data file.

Serum samples, derived from vaccinees at baseline and 21 or 70 days post influenza vaccination, were tested separately for antibodies against the three vaccine antigens (A/California/7/2009 (H1N1) pdm09, A/Texas/50/2012 (H3N2) and B/Massachusetts/2/2012 for the 1st study, and A/California/7/2009 (H1N1) pdm09, A/Switzerland/9715293/2013 NIB88 (H3N2) and B/Brisbane/9/2014 for the 2nd study. Using the HAI test, vaccine responders and non-responders were classified considering the fold increase of the titer detected on day 21 or 70 as compared to day 0. The evaluation of responses against the individual antigens revealed that in both seasons the responsiveness was considerably greater against the H3N2 antigen (1st study = 73.5%; 2nd study = 86.5 %) than against the H1N1 (58.8% and 51%) and B (32.4% and 63%) antigens (Fig. S1A)^46^. In both seasons, non-responders showed higher pre-vaccination HAI titers for all single-vaccine antigens as compared to triple responders (Table S3). In the first study, six triple non-responders (17.6%) and seven triple responders (20.6%), and in the second study, 10 triple non-responders (5%) and 71 triple responders (35.5%) were identified out of 34 and 200 vaccinees, respectively (Fig. 1). The two identified extreme groups of triple responders and triple non-responders, responding to all or none of the antigens, respectively, were used for the subsequent analyses to facilitate a clear-cut approach for assessing immune characteristics correlating with vaccine responsiveness or non-responsiveness (see Table S1 for additional background information on the cohort). The two independent study cohorts allowed the discovery and generation of a mechanistic hypothesis (1st study) and the independent validation of that hypothesis (2nd study) to identify immune mechanisms differing between responders and non-responders. The two-study design further facilitates ruling out a potential season-specific bias resulting from changes in vaccine composition, a unique feature of influenza vaccines. However, the degree of responsiveness of the volunteers to other vaccinations is unknown. Thus, it is impossible to discriminate whether putative identified defects in non-responders are influenza vaccine-specific or rather defects leading to a universal non-responsiveness to vaccination.

### Immune activation identified by serum profiling

**Fig. 2:**
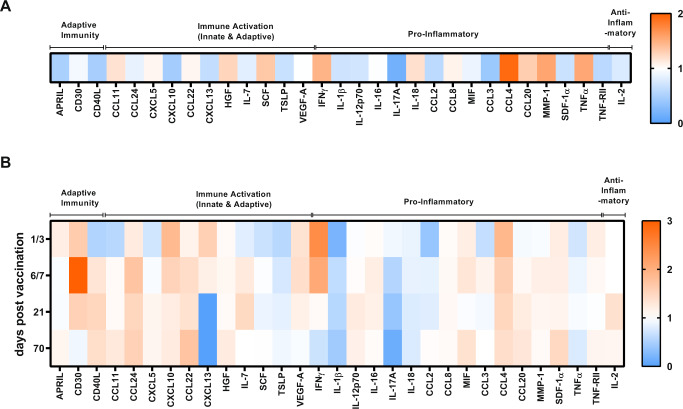
Serum profiling of vaccine responders and non-responders reveals differentially regulated factors. Cryopreserved serum samples derived from the triple responders and non-responders at all analyzed time points were subjected to a bead-based flow cytometric measurement based on Luminex technology. **A** Heat map showing the ratio of cytokine concentrations detected before vaccination in responders vs. non-responders. **B** Heat map depicting the ratio of vaccine-induced serum factors (fold increase over day 0) for triple vaccine responders as compared to non-responders. Orange represents values that show a higher response in responders as compared to non-responders, blue indicates a higher response in non-responders, 2nd study, *n* = 10 responders, *n* = 10 non-responders, biologically independent samples). Source data are provided as a Source Data file.

**Fig. 3:**
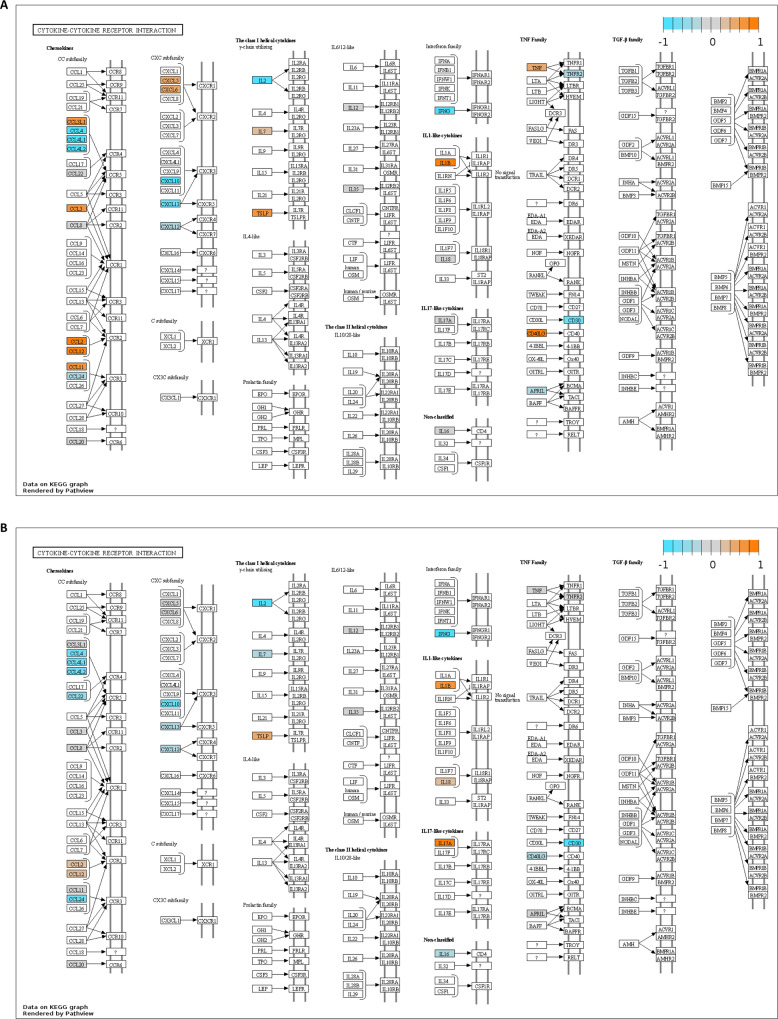
Pathway analysis of serum factors identified in samples derived from responders and non-responders (see Fig. 2). **A** 3 days post-vaccination and **B** 7 days post-vaccination (Pathview online tool). Input data = log2 values of day X/day 0. Orange indicates factors showing a higher response in responders, blue indicates factors showing a higher response in non-responders (day 3 *p* = 0.96, day 7 *p* = 0.99). *P* value calculated by GAGE.

Aiming at a broad immunological assessment of triple vaccine responders and non-responders, serum samples derived from the vaccinees before (day 0) and after vaccination (either day 1 or 3, day 7, day 21, and day 70) were subjected to bead-based flow cytometry immune profiling. In total, 65 cytokines in 165 samples (17 responders and 16 non-responders, 5 time points each) were profiled. In the context of this panel, 31 serum factors associated with the activation of innate and adaptive immune cells, inflammatory and anti-inflammatory processes could be quantified. The analyzed factors show a differential regulation in responders vs. non-responders already before vaccination (day 0) (2nd study, Fig. 2A; 1st study Fig. S1B). A direct comparison of responders vs. non-responders revealed an overall stronger induction of soluble factors in triple vaccine responders at the indicated time points post-vaccination (2nd study, Fig. 2B; 1st study Fig. S1C). Here, most identified factors are induced more strongly in responders than in non-responders, thus indicating an enhanced vaccine-induced immune activation in the responder group. All identified factors were subsequently used for a pathway analysis using the Pathview online tool. Here, an automated pathway annotation was calculated for each time point comparing vaccine responders and non-responders. The identified factors were assigned to the cytokine-cytokine receptor interaction pathway and the chemokine signaling pathway with *p* values <1 at all analyzed time points (blue = higher in non-responders, orange = higher in responders; Fig. 3A, B). Responders show a vaccine-induced activation of components belonging to the cytokine-cytokine receptor interaction pathways early (3 days) post-vaccination (Fig. 3A). The waning of this effect is apparent starting from 7 days post-vaccination (Fig. 3B). Non-responders, on the other hand, appear to maintain activation throughout all analyzed time points.

### Triple responders display solid T cell activation independent of seasonal variation

**Fig. 4:**
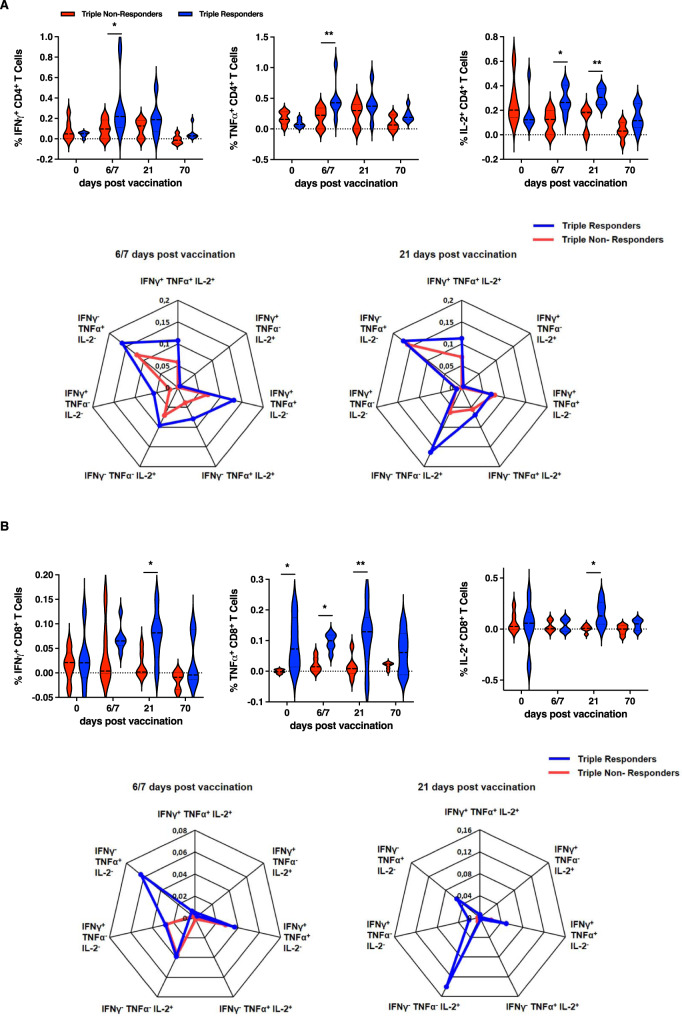
Enhanced functionality of T cell populations in vaccine triple responders. Cryopreserved PBMCs isolated from triple responders and non-responders were re-stimulated with the vaccine antigens overnight, stained for surface antigens and intracellular cytokine production and analyzed by flow cytometry. Frequencies of IFNγ^+^, TNFα^+^, and IL-2^+^ **A** CD4^+^ T cells (IFNγ day 6/7 triple non-responders vs. responders *p* = 0.0134, TNFα day 6/7 triple non-responders vs. responders *p* = 0.0092, IL-2 day 6/7 triple non-responders vs. responders *p* = 0.0130, IL-2 day 21 triple non-responders vs. responders *p* = 0.0088), **B** CD8^+^ T cells (IFNγ day 21 triple non-responders vs. responders *p* = 0.0234, TNFα day 0 triple non-responders vs. responders *p* = 0.0130, TNFα day 6/7 triple non-responders vs. responders *p* = 0.0252, TNFα day 21 triple non-responders vs. responders *p* = 0.0041, IL-2 day 21 triple non-responders vs. responders *p* = 0.0245). Violin plots represent the data subtracted for background functionality (1st study, *n* = 12 (6 non-responders and six responders, biologically independent samples, single missing values, see Source Data file). Violin plots show the mean and the quartiles as dashed and dotted lines, respectively. The shape indicated the data distribution. Asterisks denote statistical significance as calculated by Two-way ANOVA based on nominal *p* values (uncorrected Fisher’s LSD) comparing triple vaccine responders and non-responders at each time point. Source data are provided as a Source Data file.

To address the activation status and functionality of diverse T cell subsets, PBMCs were re-stimulated with a mixture of the corresponding vaccine antigens and analyzed by flow cytometry. CD4^+^ T cells derived from triple responders displayed significantly higher frequencies of IFNγ^+^ CD4^+^ and TNFα^+^ CD4^+^ T cells 6/7 days post-vaccination as compared to non-responders (1st study: Fig. 4A). Likewise, the frequency of IL-2^+^ CD4^+^ T cells was significantly higher 6/7 and 21 days post-vaccination. The analysis of multifunctional CD4^+^ T cells (producing IFNγ, TNFα, and IL-2) revealed that triple responders harbored a higher frequency of triple, double and single functional CD4^+^ T cells secreting IFNγ, TNFα, and IL-2 at all analyzed time points (1st study: Fig. 4A spider plots and Fig. S2A). The analysis of CD8^+^ T cell populations revealed significantly elevated frequencies of IFNγ-, TNFα-, and IL-2-secreting CD8^+^ T cells in triple responders as compared to triple non-responders 21 days post-vaccination (Fig. 4B). Enhanced frequencies of IFNγ- and TNFα-secreting CD8^+^ T cells in triple responders were also evident 6/7 days post-vaccination. The comparison of the vaccinees with respect to multifunctional CD8^+^ T cells (secreting IFNγ, TNFα, and IL-2) revealed no meaningful differences (1st study: Fig. 4B spider plots and Fig. S2A).

**Fig. 5:**
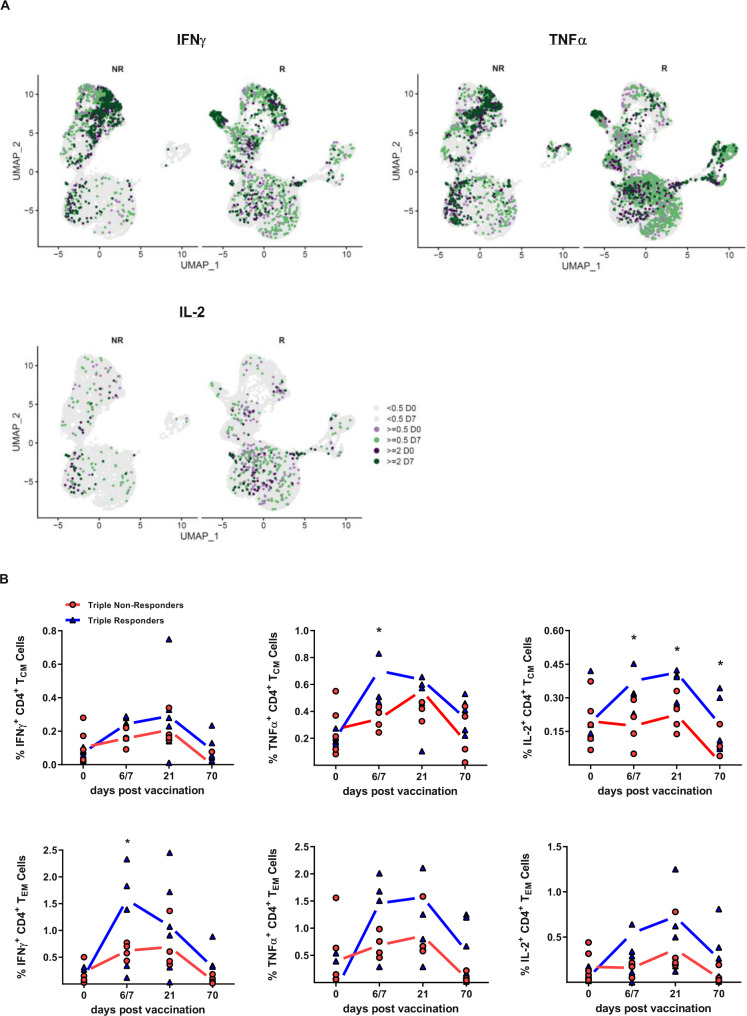
Vaccine responders show an activated T cell phenotype. **A** Single-cell RNA sequencing analysis of re-stimulated PBMCs before and 7 days post-vaccination (2nd study *n* = 6, three responders and three non-responders, biologically independent samples). The color indicates the expression density of the indicated cytokines. **B** Frequencies of IFNγ^+^, TNFα^+^, and IL-2^+^ central memory (CM, CD45RA^-^CCR7^+^) CD4^+^ T cells (TNFα day 6/7 triple non-responders vs. responders *p* = 0.0465, IL-2 day 0 triple non-responders vs. responders *p* = 0.0212, IL-2 day 6/7 triple non-responders vs. responders *p* = 0.0311, IL-2 day 21 triple non-responders vs. responders *p* = 0.04785) and effector memory (EM, CD45RA^−^CCR7^−^) CD4^+^ T cells (IFNγ day 6/7 triple non-responders vs. responders *p* = 0.0220) (1st study, *n* = 6 non-responders, and *n* = 6 responders, biologically independent samples, single missing values). Asterisks denote statistical significance as calculated by Two-way ANOVA based on nominal *p* values (uncorrected Fisher’s LSD) comparing triple vaccine responders and non-responders at each time point. Source data are provided as a Source Data file.

Data from the 2nd study confirmed the higher functional fitness of CD4^+^ T cells of responders shown by increased frequencies of IFNγ- and TNFα-secreting CD4^+^ T cells especially early after vaccination (Fig. S2C, D). Furthermore, in triple responders elevated frequencies of IL-2^+^ CD4^+^ T cells were observed 21 days post-vaccination. With regard to CD8^+^ T cell functionality, enhanced frequencies of IFNγ and IL-2-secreting cells were observed in responders as compared to non-responders 1/3 days post-vaccination (Fig. S2C, E). These functional differences between vaccine responders and non-responders were confirmed by a single-cell RNA sequencing approach (*n* = 6, three responders vs. three non-responders, 2nd study). In non-responders, IFNγ secretion is mainly confined to the identified NK cell cluster at all analyzed time points, whereas responder-derived cells show a strong vaccine-induced IFNγ secretion additionally within various CD4^+^ and CD8^+^ T cell clusters with a special focus on CD8^+^ T_CM_ cells (Fig. 5A). A similar pattern is observed for TNFα-secreting cells. In the non-responders, most of these cells are found in the NK and some CD4^+^ T memory (T_M_) cell clusters. Strikingly, in the responders, these clusters only harbor few TNFα-secreting cells, whereas naïve and T_M_ cells show a robust activation. IL-2 secretion is quite low in non-responders and higher mainly within the CD4^+^ T cell clusters in the responders.

The observed functional discrepancy between CD4^+^ T cell subsets analyzed from vaccine responders and non-responders is mirrored by the frequencies of T_H_1, T_H_2, and T_H_17 cells according to the expression of CXCR3 and CCR6 (Fig. S2B). The T_H_1 (CD4^+^CXCR3^+^CCR6^-^) and T_H_2 (CD4^+^CXCR3^−^CCR6^−^) cell populations derived from vaccine responders but not from non-responders expand upon vaccination, peaking 6/7 days after the immunization, returning later to baseline levels. In contrast, significantly lower frequencies of T_H_17 (CD4^+^CXCR3^-^CCR6^+^) cells were observed in responders with respect to non-responders at early time points in both studies (day 0 and 6/7 post-vaccination).

The stimulation/recall of antigen-specific memory CD4^+^ T cells, derived from previous influenza infections or vaccinations, was assessed by the frequencies of cytokine-secreting central memory (CM) and effector memory (EM) T cell populations (CD45RA^-^ CCR7^+^ and CD45RA^-^ CCR7^-^, respectively). Significantly higher frequencies of IFNγ^+^ and TNFα^+^ CD4^+^ T_CM_ cells were detected in triple responders than in triple non-responders 6/7 days post-vaccination (1st study, Fig. 5B). Triple responders also displayed significantly elevated frequencies of IL-2^+^ CD4^+^ T_CM_ cells 6/7, 21 and 70 days post-vaccination. The functional analysis of CD4^+^ T_EM_ cells revealed significant differences regarding IFNγ-producing CD4^+^ T cells on day 6/7 post-vaccination. Higher frequencies were found in triple responders, whereas TNFα- and IL-2-secreting CD4^+^ T_EM_ cells were only marginally increased (Fig. 5B). The results gained from the 2nd study confirm this overall higher functionality of central and effector memory cells derived from triple vaccine responders (Fig. S2F). These data clearly depict that triple responders not only develop a stronger initial T cell response following influenza vaccination, but also an enhanced vaccine-induced antigen-specific memory response as compared to vaccine non-responders.

The senescence of immune cell populations in the elderly is described as crucially affecting their functional properties. Interestingly, the frequency of senescent CD4^+^ and CD8^+^ T cells did not differ between triple vaccine responders and non-responders before or after vaccination (Fig. S3A 1st study and S3B 2nd study). The functionality of senescent CD4^+^ and CD8^+^ T cells, however, differed between triple vaccine responders and non-responders, showing an overall enhanced vaccine-induced functionality (IFNγ and TNFα secretion) in triple responders, as compared to non-responders. These findings highlight that an enhanced functionality of T cell populations derived from triple vaccine responders is not only inherent to effector cells, but also to senescent CD4^+^ and CD8^+^ T cells. Thus, the progression of functional, but not phenotypic immune senescence, might differentiate vaccine responders and non-responders.

### High frequencies of regulatory T cells correlate with poor vaccine responsiveness

**Fig. 6:**
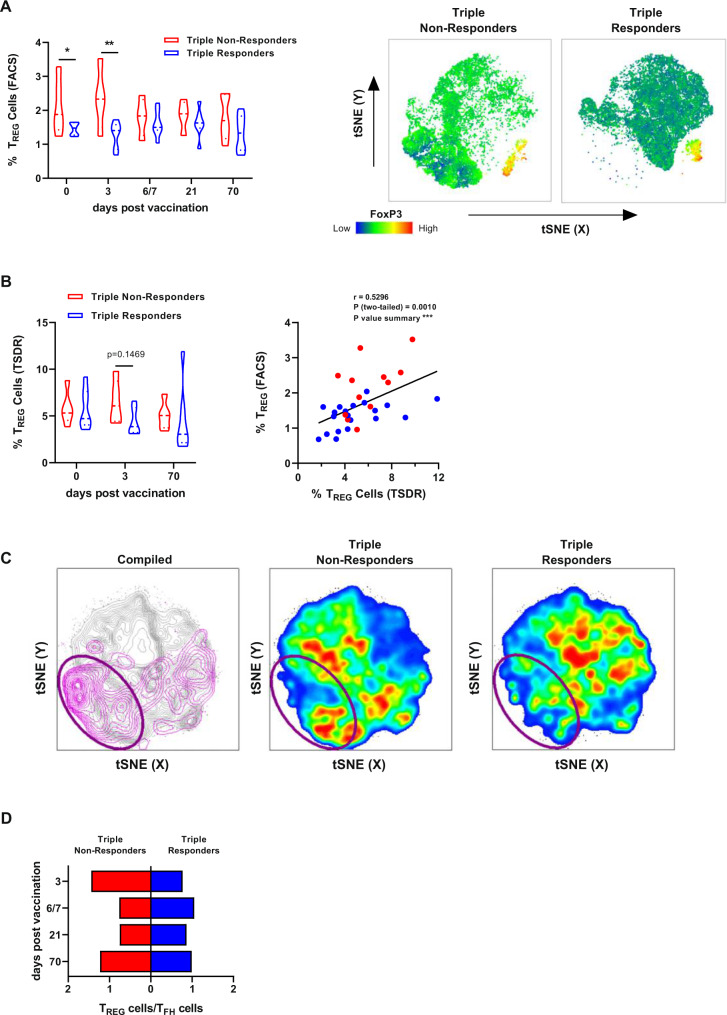
T_REG_ and T_FH_ cells as important factors for vaccine-induced immune responses. Cryopreserved PBMCs isolated from vaccine triple responders and non-responders were stained for surface antigens and intranuclear expression of FOXP3 ex vivo and analyzed by flow cytometry. **A** Frequencies of T_REG_ cells (CD4^+^CD127^low^CD25^+^FOXP3^+^) assessed by flow cytometry (1st study, *n* = 5 and *n* = 7 non-responders and responders, respectively, biologically independent samples, single missing values, see Source Data file, day 0 triple non-responders vs. responders *p* = 0.0390, day 6/7 triple non-responders vs. responders *p* = 0.0030). Plots show representative tSNE analyses of triple non-responders and responders with the expression density of FOXP3 highlighted (blue = low expression, red = high expression). **B** Frequencies of T_REG_ cells assessed by TSDR analysis and correlation with T_REG_ cells assessed by flow cytometry before, 3 and 70 days post-vaccination (1st study, *n* = 5 and *n* = 7 non-responders and responders, respectively, biologically independent samples, Spearman correlation). **C** Plot (left) depicting the tSNE analysis of 4 triple non-responders and 4 triple responders (gray) with manually gated T_REG_ cells highlighted and gated accordingly (CD3^+^CD4^+^FOXP3^+^ = purple). tSNE plots of non-responders and responders depicted in pseudo colors indicating low (blue) and high (red) cell densities 3 days post-vaccination. **D** Mean values of T_REG_ and T_FH_ cell frequencies at the indicated time points post-vaccination normalized to day 0 (1st and 2nd study, *n* = 10 non responders and *n* = 15 responders, biologically independent samples). Violin plots show the mean and the quartiles as dashed and dotted lines, respectively. The shape indicated the data distribution. Asterisks denote significant nominal *p* values as calculated by two-way ANOVA or Mixed-effects analysis for data sets with missing single values (uncorrected Fisher’s LSD, see Source Data file) comparing triple vaccine responders and non-responders at a given time point Source data are provided as a Source Data file.

T_REG_ cells play a crucial role by controlling the activity of a wide variety of immune cells. To assess their potential contribution to the observed differences in responsiveness, cryopreserved PBMC samples derived from responders and non-responders were analyzed ex vivo for the frequency and absolute cell number of T_REG_ cells. At baseline as well as 3 days post-vaccination, triple vaccine non-responders harbored significantly more T_REG_ cells (CD4^+^CD127^low^CD25^+^FOXP3^+^) as compared to triple vaccine responders (1st study, Fig. 6A). This tendency remains stable at all assessed time points. The analysis of the tSNE further highlights the differences in the fingerprints of the T_REG_ cell population in responders and non-responders. The assessment of the absolute T_REG_ cell numbers supports a higher number of vaccine non-responders at early time points (1st study, Fig. S4A). However, the identification of T_REG_ cells by flow cytometry is controversially discussed with regard to the applied gating strategy^47,48^. Thus, we performed an additional analysis for the unequivocal identification of T_REG_ cells, the assessment of the methylation state of the T_REG_ cell-specific demethylated region (TSDR), which confirmed the higher T_REG_ cell frequencies in triple non-responders as compared to responders 3 days post-vaccination (1st study, Fig. 6B). The values derived from TSDR and FACS analyses show a significant correlation. The PBMC samples from the 2nd study further confirmed this association by flow cytometry as well as TSDR analysis, since enhanced frequencies of T_REG_ cells were also observed in non-responders 3 days post-vaccination (Fig. S4B). Thus, the reduced functionality of CD4^+^ and CD8^+^ T cells in triple non-responders might be ascribed to higher frequencies of T_REG_ cells. A tSNE analysis of samples derived from responders and non-responders 3 days post-vaccination distinctly showed differences in the T_REG_ cell compartment (Fig. 6C). The compiled plot shows all CD3^+^CD4^+^ T cells with the CD127^low^CD25^+^FOXP3^+^ T_REG_ cell population highlighted in purple. The purple gate comprises T_REG_ cells as indicated in the compiled plot, highlighting the differences between responders and non-responders. T_FH_ cells represent an additional critical factor for the stimulation of efficient humoral responses after vaccination. Frequencies of T_FH_ cells were by trend higher in responders than in non-responders at all analyzed time points (Fig. S4A 1st study and B 2nd study). The mutual evaluation of T_REG_ cells and T_FH_ cells was performed by calculating the ratio of T_REG_/T_FH_ using values normalized to day 0, thus giving a ratio of the vaccine-mediated impact. Here, T_REG_ cells dominated the response in triple non-responders 3 days post-vaccination, whereas the T_FH_ increase was higher in responders at this time point (Figs. 6D, S4C). At later time points, these ratios shifted with an increased T_REG_ response in responders, leading to a convergence of ratios 70 days post-vaccination. These data highlight the presence of a suppressive immune phenotype in non-responders at baseline and early after vaccination that might account for the hampered responsiveness.

### Mathematical modeling of vaccine-induced T cell responses

**Fig. 7:**
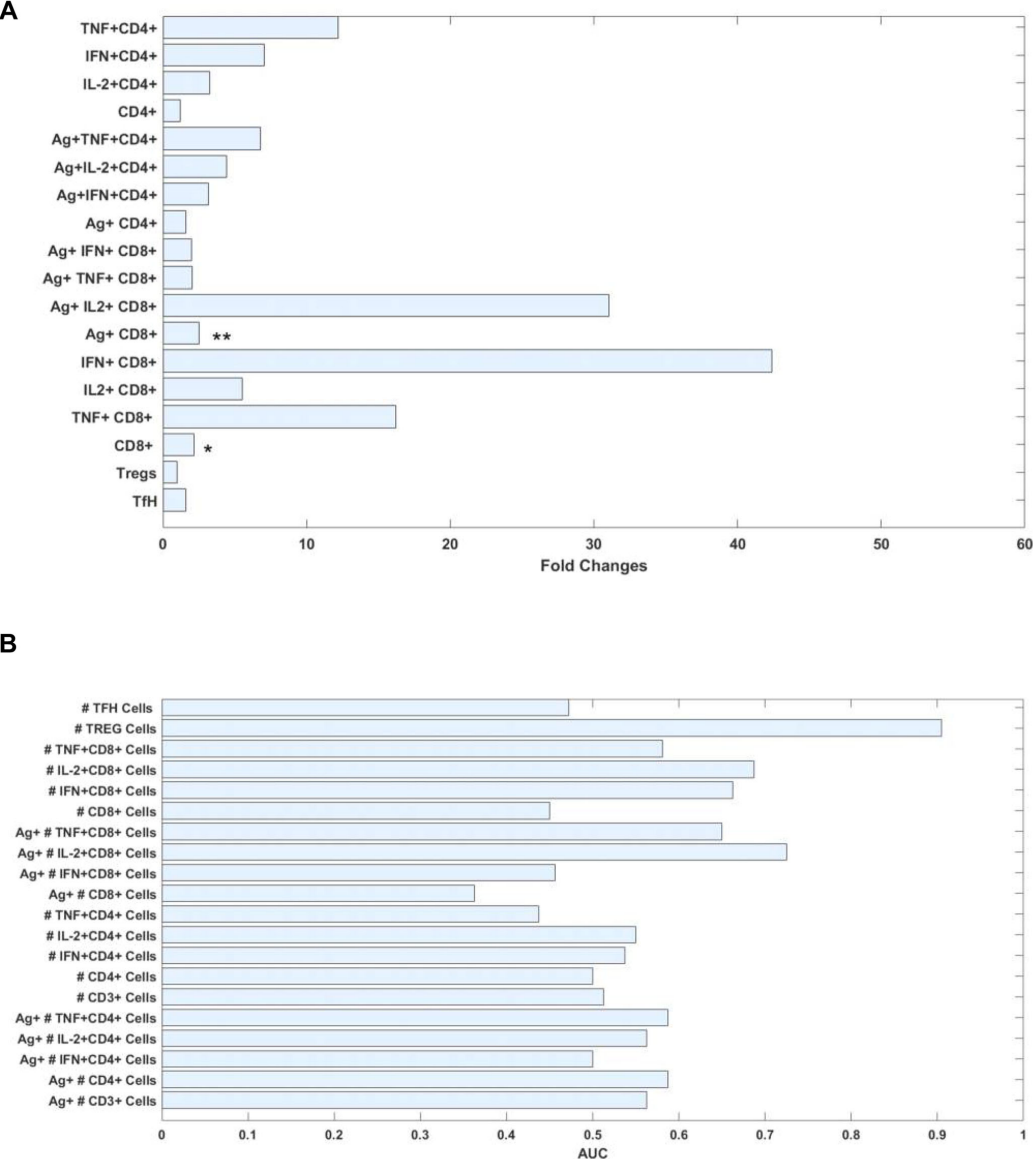
Predictive potential of data on T cell functionality. Data derived from the flow cytometric analysis was subjected to mathematical modeling approaches. **A** A linear model to compute the expansion rates (cells/day) during the first week post-vaccination was computed in responders and non-responders. The bars represent the average fold change increase in expansion rates of responders with respect to non-responders (Ag^+^CD8^+^ *p* = 0.002, CD8^+^ *p* = 0.049). **B** Considering a multiclass naïve Bayes model, the AUC is presented to highlight marker candidates to differentiate between responders vs. non-responders. Asterisks denote significant values as calculated by a two-sample two-tailed t-test and a two-tailed Wilcoxon rank sum test comparing triple vaccine responders and non-responders at the indicated time point.

A mathematical model to represent the “expansion” of cell compartments, describing vaccine-induced kinetic changes with regard to their population size, was used to compute a combination of different events happening in the blood compartment 7 days post-vaccination e.g., proliferation, migration, and differentiation. The modeling analysis of data from the 2nd study derived from the first week post-vaccination revealed that triple responders present expansion rates of cell populations (cells/day) higher than triple non-responders within different cell sub-compartments (at least two folds higher, Fig. 7A). In particular, the expansion of the CD8^+^ T cell compartment (stimulated and unstimulated) is significantly higher in the triple responders. Interestingly, although not statistically significant, the only cell compartment exhibiting a higher cell expansion in the triple non-responders were T_REG_ cells, possibly explaining the observed limited cell expansion rates as well as the described reduced functionality. Data derived from the first study is not shown due to insufficient statistical power.

In the search for early predictors distinguishing triple responders and non-responders, we further employed the area under the curve (AUC) for ROC curves based on the classic logistic model as well as a multiclass naïve Bayes model. During the first week post-vaccination, T_REG_ cells (CD3^+^CD4^+^FOXP3^+^) harbored the highest predictive value (AUC approximately 0.9) with respect to the other cell compartments, thus substantiating the hypothesized role of T_REG_ cells as a driving force that determine influenza vaccine responsiveness (Fig. 7B).

### Impaired memory B cell responses confirm hampered humoral immunity in vaccine non-responders

**Fig. 8:**
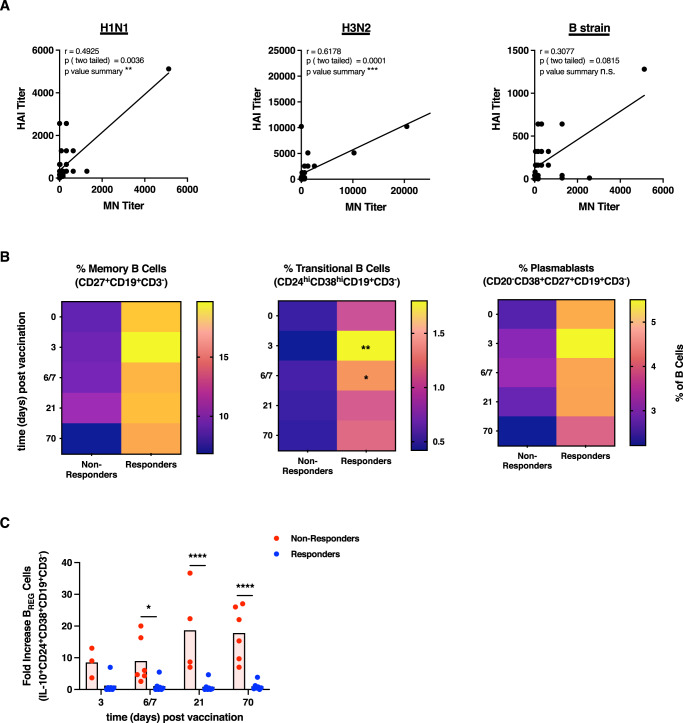
B cell responses in vaccine responders and non-responders. Frozen sera derived from vaccinees 21 days after vaccination in the two seasons were used for quantification and qualification of humoral immune responses by HAI and MN assays, respectively. **A** Correlation of the HAI and MN titer. The shown data is derived from the 1st and the 2nd study (*n* = 33 biologically independent samples). Asterisks denote significant relationships as calculated by Spearman correlation (two-tailed). Cryopreserved PBMCs isolated from vaccine triple responders and non-responders were stimulated with the vaccine antigens and stained for surface antigens identifying **B** memory B cells (CD27^+^CD19^+^CD3^-^), transitional B cells (CD24^hi^CD38^hi^CD19^+^CD3^-^, day 3 triple non-responders vs. responders *p* = 0.0039, day 6/7 triple non-responders vs. responders *p* = 0.0386) and plasma blasts (CD20^-^CD38^+^CD27^+^CD19^+^CD3^-^). Heat maps show the means of frequencies (% of CD19^+^CD3^-^ B cells) of re-stimulated samples (2nd study, *n* = 6 non responders and *n* = 7 responders, biologically independent samples). **C** Fold increase of B_REG_ cells (IL-10^+^CD24^+^CD38^+^CD19^+^CD3^-^) at the indicated time points as compared to day 0 (2nd study, *n* = 6 non responders and *n* = 7 responders, biologically independent samples, day 6/7 triple non-responders vs. responders *p* = 0.0199, day 21 triple non-responders vs. responders *p* < 0.0001, day 70 triple non-responders vs. responders *p* < 0.0001). Columns represent the mean ± SEM of data from re-stimulated samples with individual values depicted as dots. Asterisks denote significant values as calculated by Two-way ANOVA without correction for multiple comparisons (uncorrected Fisher’s LSD) comparing triple vaccine responders and non-responders at a given time point. n.s. = not significant. Source data are provided as a Source Data file.

The functional relevance of antigen-specific antibodies used to stratify the vaccinees was confirmed by the qualitative assessment of antibody functionality according to the microneutralization (MN) titer (1st and 2nd study, Fig. 8A). A significant positive correlation of the HAI and the MN titers was found for the H1N1 and H3N2 strains contained in the vaccine formulation, as well as a clear trend for the B strain. As for pre-vaccination HAI titers, pre-vaccination MN titers were in general higher in triple non-responders (Table S3). Regarding the B cell populations contributing to antibody secretion, the memory and transitional B cells as well as plasmablasts were assessed by flow cytometry upon antigen re-stimulation. Triple-vaccine responders showed significantly higher frequencies of all three cell types as compared to non-responders (Figs. 8B, S5A). Herein, significant differences were detected for transitional B cells, 3 and 6/7 days post-vaccination as compared to baseline. These findings confirm the severe deficiencies in the humoral compartment in vaccine non-responders. In addition to the described suppressive phenotype in non-responders shaped by enhanced frequencies of T_REG_ cells, significantly increased frequencies of regulatory B (B_REG_) cells were also found in non-responders but not in responders upon vaccination (2nd study, Fig. 8C; 1st study, Fig. S5B). The differences were most striking at later time points post-vaccination. Overall, these results suggest that impaired antibody responses might be shaped by both suppressive regulatory T and B cells.

### Identification of different transcriptomic fingerprints in vaccine responders and non-responders

The above data distinctly showed increased cellular and humoral functionality in vaccine triple responders, whereas triple non-responders harbored a rather suppressive phenotype characterized by enhanced frequencies of T_REG_ and B_REG_ cells. To gain a comprehensive view of functional differences on the transcriptional level, single-cell RNA sequencing experiments were performed. For this, cryopreserved PBMCs isolated from 6 selected triple responders and non-responders vaccinated in the 2nd study were re-stimulated with a mixture of the vaccine antigens and subsequently analyzed. In total, transcriptomes of PBMCs derived from 12 samples (3 responders and 3 non-responders at day 0 and day 7) were profiled.

**Fig. 9:**
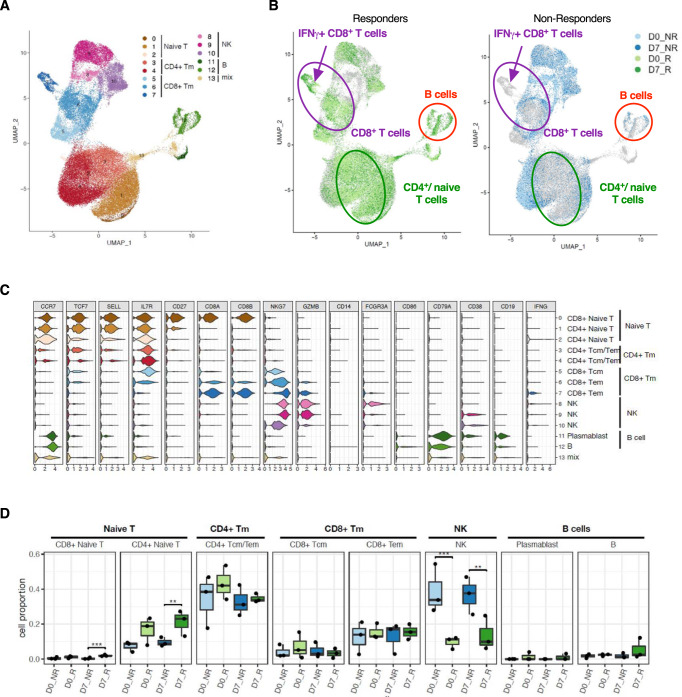
Single-cell RNA sequencing of samples derived from vaccine responders and non-responders. Cryopreserved PBMCs were re-stimulated with the vaccine antigen mixture and subjected to sorting for CD45^+^ cells prior to further measurements. **A** Identified clusters and **B** comparison of cells derived from responders and non-responders according to the time point of sample drawing (light green/blue = responders/non-responders day 0, dark green/blue = responders/non-responders day 7 post-vaccination). **C** Gene signatures identifying the cells within the clusters. **D** Cell abundancies (rel. counts [%]) of identified cell populations, significant changes between responders and non-responders at either D0 or D7 were marked. *n* = 6 (three responders (R)/three non-responders (NR), at baseline (D0) and 7 days post (D7) vaccination, biologically independent samples). The lower and upper hinges of the box plots correspond to the first and third quartiles (the 25th and 75th percentiles). The central line represents the second quartiles (the 50th percentiles). The upper whisker extends from the hinge to the largest value. The lower whisker extends from the hinge to the smallest value. Statistically significant differences were calculated by Dirichlet-multinomial regression adjusted with the Benjamini–Hochberg method (CD8^+^ naïve T cells day 7 triple non-responders vs. responders *p* = 0.0001, CD4^+^ naïve T cells day 7 triple non-responders vs. responders *p* = 0.005, NK cells day 0 triple non-responders vs. responders *p* = 0.0001, NK cells day 7 triple non-responders vs. responders *p* = 0.0047).

The annotation of cell clusters identified naïve CD4^+^ and CD8^+^ T cell populations, including memory subsets, B cells as well as NK cells according to the expression of signature genes (Fig. 9A, C). The comparison of data derived from responders (R) and non-responders (NR) before (D0, light green/blue) and 7 days post-vaccination (D7, dark green/blue) clearly revealed different patterns and expression profiles (Fig. 9B, Fig. S6A, B). Cell clusters ascribed to CD4^+^ as well as CD8^+^ T cells, e.g., IFNγ^+^ CD8^+^ T cells, as well as B cell populations, e.g., CD38^+^ CD19^+^ plasma blasts, were predominant in responders (Fig. 9C). The analysis of the relative cell counts (rel. counts [%]) revealed significantly elevated numbers of naïve CD8^+^ and CD4^+^ T cells in responders at day 7 as compared to non-responders (Fig. 9D). Interestingly, profound differences were also observable for NK cells with significantly higher counts detected in non-responders at both time points.

**Fig. 10:**
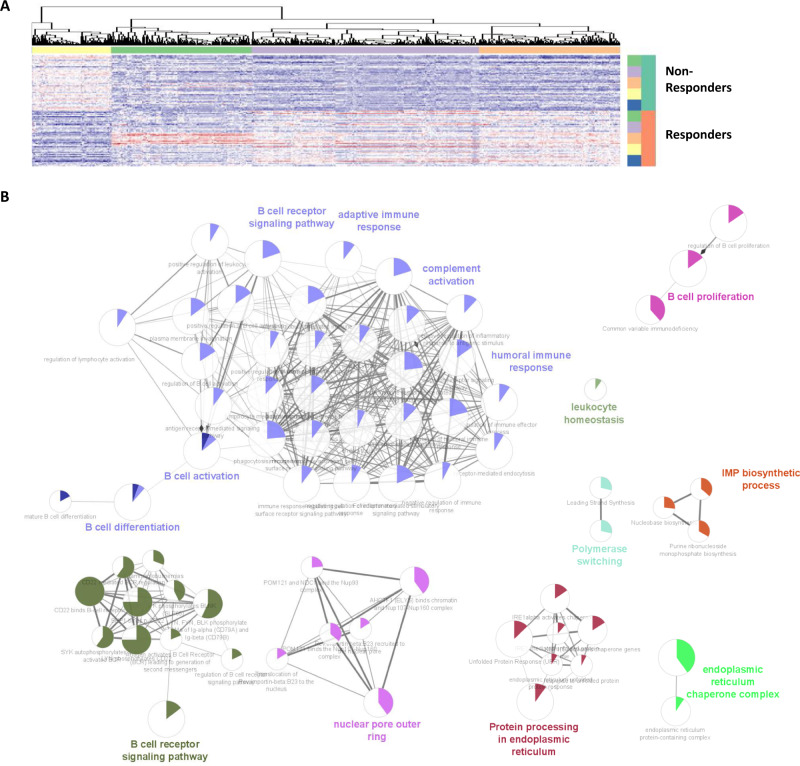
Functional analysis of differentially expressed genes in responder transcriptomes (bulk genome sequencing). **A** The heatmap displays the top 500 ranked differentially expressed genes among responders (*n* = 10 biologically independent samples) and non-responders (*n* = 10 biologically independent samples) at different time points (V1=green, V2=purple, V3=orange, V4=yellow, V5=blue) based on adjusted *p* values (Benjamini-Hochberg). A generalized linear model (GLM) is applied to detect statistical differences at gene level. A z-score based color range (red-white-blue) is used to show relative gene expression (high – median – low). Genes showing similar expression profiles are clustered into five gene sets (horizontal color bars). **B** Each gene set is applied to gene set enrichment analysis (GSEA) using the following databases: GO, KEGG and Reactome. Only pathways (terms) with corrected *p* values below 0.05 are displayed. Terms are connected among each other to create a larger module based on their functional known interrelations using ClueGo app (part of cytoscape visualization tool)^116,117^. Color-filled sections in circles (terms) represent the ratio of genes detected and genes belonging to the corresponding term.

To expand the findings of the scRNA analysis, a bulk RNA-seq analysis of whole blood, taken at baseline and 1/3, 7, 21, and 70 days (V1–V5, respectively) post-vaccination was performed to identify differentially expressed genes (DEG) discriminating vaccine responders and non-responders (Fig. 10A, B, Fig. S6C, D). A total of 664 genes were differentially expressed. These can be further grouped into six clusters of co-regulated genes according to their appearance after time (Fig. 10A). Gene set enrichment analysis (GSEA) using GO-Terms “Biological Process” revealed that critical immune processes are becoming more activated in responders, like “B cell receptor signaling pathway”, “Intestinal immune network for IgA production”, “Protein processing in endoplasmic reticulum”, “Cell cycle”, “T cell activation” and “B cell activation” (Fig. 10B).

In addition, a comparison of the fecal microbiota composition in the groups of triple responders and non-responders was performed using 16S rRNA analysis of the V4 region. No specific clustering during analysis of β-diversity and a similar α-diversity between the groups was observed (Fig. S8A, B). Furthermore, a similar abundance of all bacterial families present in the intestinal community of the investigated participants was detected (Fig. S8C).

## Discussion

Despite availability of vaccines tailored for an aged immune system, unacceptably high percentages of influenza vaccine non-responders are still observed in the elderly. This bottleneck will persist even for the foreseeable next generation of universal vaccines. In fact, this roadblock goes beyond aspects related to the identification of antigens conferring broader responses, immune senescence, and immune imprinting. Why do certain individuals within the same demographic group, sharing similar antigenic challenges and health status respond to vaccination whereas others do not? What are the key commonalities and differences? This knowledge gap on the underlying mechanisms represents the true roadblock to the development of vaccines against influenza and other pathogens tailored for the elderly. Using a two-seasonal clinical research approach, which combines serological, flow cytometric and genomic readouts coupled to a bioinformatics analysis, we contribute to answering these questions.

To gain a global unbiased understanding of the mechanisms dictating vaccine efficacy independently of age-related immunological shortcomings, an in-depth immune profiling of elderly responding or not to vaccination is indeed crucial. With regard to influenza vaccination, it is essential to keep in mind that vaccines need to be adapted from season to season due to viral evolution. This represents a major hurdle for the implementation of systems vaccinology approaches and/or the establishment of the significance of a hypothesis derived from clinical studies. Thus, to assess the universal value of identified mechanisms or predictive nodes, it is critical to validate hypotheses or candidate targets derived from one study using samples obtained from a comparative cohort vaccinated in another season. Thus, our 1st study was established as a test bed to identify putative players and to generate hypotheses on potential mechanisms, whereas the 2nd study was structured to validate those hypotheses using a larger cohort. This approach led us to an overall conclusion: non-responsiveness is characterized by a suppressive immune phenotype. The overarching regulatory framework and interconnected nodes emerging from our study offer several molecular intervention targets to adapt, refine and tailor vaccination strategies for the elderly.

The classification of vaccinees into polar groups of triple responders and triple non-responders that facilitated the subsequent analysis was carried out according to the EMA guidelines. Although the vaccine used was especially approved for use in the elderly, the response rates were comparatively low. In both seasons, approximately one-third of the vaccinees responded to none or only one of the vaccine antigens, demonstrating that even a vaccine specifically tailored to the elderly can provide a false perception of the level of conferred protection.

Vaccine efficacy depends on the individual responsiveness of the vaccinees, highlighting the importance of individualizing or at least stratifying approaches targeted to poor responders in high-risk groups^49^. Age, gender, underlying co-morbidities, co-infections, vaccination history, and influenza immune imprinting are described as key factors biasing vaccine-induced immunity. In fact, preexisting immunity to influenza is one major determinant of vaccine responsiveness^50–52^. Immunological imprinting by the first influenza viruses encountered by an individual appears to be a major driving force for antibody responses against drifted or new viruses or vaccine antigens^53,54^. In this regard, influenza infections encountered in childhood permanently shape responses, thereby affecting the outcome of vaccinations later in life. Consequently, all vaccinations later in life are indeed booster vaccinations and have to be assessed accordingly. Our study of a more demographic homogeneous elderly group contributes to reduce biases caused be previous encounters. Interestingly, individuals stratified as non-responders received influenza vaccinations more frequently in previous years as compared to responders (information derived from medical questionnaire, see Table S1). This is in line with a systemic meta-analysis reporting that repeated vaccination attenuates vaccine effectiveness^55^. According to the theory of antigenic sin, vaccination history might contribute to differences in vaccine responsiveness. Therefore, the vaccination history of each individual could be one of the parameters to be taken into account for the stratification of vaccinees. Furthermore, co-morbidities (e.g., obesity/metabolic dysfunction, heart or lung diseases) can also affect responsiveness to vaccination^56–61^. Here, our data showed that self-reported diabetes is associated with reduced humoral immunity to influenza vaccination^46^. This personal immunological baggage is particularly heavy in aged individuals, like those profiled here.

The implementation of multilayer integration strategies to dissect complex processes is facilitated by continuous technological advancement^62^. Systems biology-driven approaches combining multi-omics big data coupled to bioinformatics and artificial intelligence emerged as key tools for unbiased identification of the involved nodes^63,64^. The combination of immunologic, genetic, and transcriptional data allowed identification of host genetic factors contributing to influenza vaccine responsiveness^65^, as well as generating a computational framework for predictive models^66^. Thus, we selected this powerful unbiased approach rather than a hypothesis-driven one. In fact, most knowledge on vaccine-induced immunity in the elderly is derived from mouse models, but the direct translation of these results to humans is disputable. The aging process of mice does not directly reflect the one of humans, particularly considering the different life spans. Furthermore, the comorbidities and medications found in elderly humans, which crucially impact the immune system, cannot be appropriately modeled in mice. The same is true for other aspects, such as influenza immune imprinting and genetic variation. In this regard, naïve rather than pre-infected mice are frequently used for research studies, although primed mice would more closely resemble the situation of the elderly, who were exposed multiple times to the influenza virus and vaccines during their life. This crucially relevant knowledge gap was specifically addressed in our study, by immune profiling of well-defined human cohorts.

The comparison of the soluble mediators present in serum samples derived from vaccine triple responders and non-responders showed a differential activation of signaling pathways associated with innate and adaptive immune activation as well as pro- and anti-inflammatory responses, e.g., activation of cytokine receptor signaling and chemokine signaling. Before vaccination, non-responders showed a strong, presumably unspecific immune activation that might account for the observed dampened vaccine-induced T-cell functionality. The resulting lower vaccine-induced immune activation is in line with earlier findings describing that reduced humoral responses correlate with reduced T cell functionality in the elderly, especially IFNγ secretion, upon vaccination against Japanese encephalitis^67^. In this regard, the efficient activation of T cells in the elderly after vaccination requires approaches that aim at promoting T cell functionality in the context of a senescent immune system, such as incorporation of suitable adjuvants^68,69^. The MF59C.1-adjuvanted influenza vaccine used in this study (Fluad) represents one of such approaches, and was shown to efficiently reduce influenza-related severe disease outcomes among the elderly^8,11^. The different frequencies of T_REG_ and T_FH_ cells detected in responders and non-responders before and early after vaccination point towards a repressive immune response in non-responders and highlight the potentially crucial role of these cell populations for vaccine efficacy. Thus, their interplay appears as an important determinant of vaccine responsiveness in the elderly. With regard to influenza vaccination, T_REG_ cells were shown to participate in the downregulation of vaccine-induced antibody responses in healthy adults and it was proposed that their modulation can improve vaccine responsiveness^70^. This observation is in line with earlier findings showing that T_REG_ cells are responsible for a poor response in cancer and HIV vaccination trials^71^. In agreement with this, an explorative biomarker study after vaccination of middle-aged adults with a tetravalent meningococcal vaccination showed that higher pre-vaccination frequencies of naïve CD4^+^ T and T_REG_ cells correlate with poor vaccine responsiveness, thereby rendering T_REG_ cells as potential early biomarkers for vaccine responsiveness^72^. Suppression of T_REG_ cell responses in predicted vaccine non-responders might facilitate efficient immune activation, resulting in improved vaccine-induced immunity. In this line, therapeutic inhibition of T_REG_ cell functionality has been described for the treatment of oncological malignancies^73,74^. Interestingly, T_FH_ cells were reported as predictors for influenza vaccine-induced antibody responses in adults but not in the elderly^75^. However, this study compared samples derived from adults and elderly individuals, whereas differences between responders and non-responders within the elderly were not assessed. Thus, an algorithm based on the number, functional properties, and ratios of T_REG_ and T_FH_ cells might represent an improved predictor for vaccine-induced immunity, especially but not exclusively in the elderly. Unfortunately, the performed transcriptomic bulk and single-cell analyses did not yield a sequencing depth allowing an assessment of T_REG_ and T_FH_ cells, which was due to the low abundancies of these cell populations.

The assessment of B cells further contributed to understanding the functional differences between vaccine responders and non-responders. A lack of memory populations, especially plasmablasts, together with enhanced numbers of suppressor B_REG_ cells characterize the fingerprint of non-responders. This is in accordance with the detected impaired immune responses. Plasmablasts are critical for recall responses and reduced quantities of vaccine-specific antibodies are the major reason for poor vaccine efficacy^76,77^. Regulatory T and B cells were described to negatively impact cellular responses to the pandemic H1N1 influenza vaccine in HIV-infected children and youths^78^. Thus, although regulatory responses are essential for a balanced and efficient immunity, the enhanced frequencies of B_REG_ cells detected in non-responders upon vaccination might further contribute to an overall suppressive immune phenotype.

Vaccine-non-responsiveness amongst the elderly will also be strongly affected by immune senescence^67,79–81^. Higher frequencies of senescent T cells commonly correlate with reduced vaccine efficacy in studies in which immune function in elderly and young adults was compared^82^. However, a differential advancement of phenotypic senescence, as detected by means of CD28 and CD57, does not correlate with vaccine responsiveness in this study. This is in contrast with an earlier publication describing an association of enhanced CD8^+^CD28^null^ cell frequencies with a poor responsiveness to influenza vaccination in the elderly^83^. However, the use of a non-adjuvanted vaccine (Vaxigrip) in the reported study might in part explain the differences. On the other hand, the functional analysis of senescent T cells confirmed the existence of functional differences between vaccine responders and non-responders with the former showing a higher cytokine secretion upon vaccination. The secretion of cytokines by senescent T cells is not surprising, since the markers CD28^-^CD57^+^ are used to identify cells showing replicative senescence rather than exhaustion^24^. In fact, differential replicative senescence or functional exhaustion in influenza vaccine responders and non-responders seems to contribute to vaccine efficacy^84^. Furthermore, the observed differences in functionality might represent one facet of the immune senescence process, thus indicating a higher degree of immune senescence in the non-responders as compared to the responders. A similar observation was made in the context of an elderly influenza vaccination cohort, where incomplete vaccine responders showed signs of accelerated T cell aging^85^. However, it would be important to gather information on responsiveness to other vaccines in order to dissect general vs. vaccine-specific effects. Furthermore, vaccination history appears as a critical discriminating parameter between influenza vaccine responders and non-responders. This points towards the theory of antigenic sin (i.e., immune imprinting) as an additional factor. Regardless of the underlying mechanisms behind non-responsiveness, it is still important to identify potential intervention targets to improve vaccine efficacy in this subpopulation. Underlying chronic inflammation might additionally contribute to a differential functional senescence in responders and non-responders. In the elderly, a higher rate of chronic inflammation is common and its impact on the immune system is termed inflammaging^86^. In this context, the impact of persistent cytomegalovirus (CMV) infections on vaccine efficacy is controversially discussed^87–91^. In the presented study, no correlation between CMV sero-status and vaccine responsiveness was observed^46^. Nevertheless, CMV is not the only chronic infection commonly found in the elderly, and vaccine responsiveness could be affected by any of them. The individual medical history and medications, as well as the nutritional status and the microbiome might additionally skew the analyzed immune responses^92–95^. However, here, the microbiome of vaccine triple responders and triple non-responders did not show any significant differences that might account for the differential responsiveness, with no clustering, and similar diversities and abundances of bacterial families.

As an additional determinant, gender-specific differences in the elicited immune responses should be considered. In the 1st study, female participants dominated the responder group (five out of six individuals), whereas amongst the non-responders both genders were equally represented. In the 2nd study, both groups were nearly equal in composition. In general, females show stronger antibody responses, more adverse effects, and a higher vaccine efficacy upon influenza vaccination as compared to male vaccinees^96–98^. These differences are mainly ascribed to the effects exerted by the sex hormones. Binding of steroids to their receptor directly influences the secretion of cytokines as well as T and B cell functionality. While testosterone dampens immune responses against influenza, estradiol can stimulate antibody production^97^. However, as estradiol levels decrease with age, this effect is described to be diminished^99^. These differences, described for a variety of vaccines besides influenza (e.g., BCG, MMR, and yellow fever), highlight that immunization approaches should consider very carefully the gender-factor^100^.

Despite all the listed unknown variables individually affecting immune responses, the described analyses distinctly highlight functional differences with regard to cellular and humoral immune responses in vaccine responders and non-responders in the elderly group. However, the scRNA seq results clearly show that, although all responders display better functionality, the responses are individually different. Out of the analyzed responders, each shows an individual functional fingerprint, thereby highlighting that the limited effector mechanisms defining vaccine responsiveness (i.e., HAI or MN titers) do not result from a uniform or identical immune response pattern, but rather from the integration of a complex, pleomorphic and individual fingerprint. Nevertheless, the detailed and comprehensive analysis of non-responders revealed a potential overarching intervention point for future vaccine designs: the key regulatory modules responsible for immune contraction and limitation of overshooting responses. Partial attenuation of these mechanisms appeals as a promising approach to promote protective immune responses in poor responders after vaccination. Considering the dynamics of the observed differences, assessment of T_REG_ and T_FH_ cell frequencies and/or related functional biomarkers at baseline or early post-vaccination could contribute to a rapid identification of vaccine non-responders.

## Methods

The performed study complies with all relevant ethical regulations. The execution of the study as well as the use of the sampled human material was approved by the ethics committee of Hannover Medical School. The documentation of all findings adheres to the Strengthening the Reporting of Observational Studies in Epidemiology (STROBE) reporting guideline for cohort studies.

### Study population and sample preparation

We conducted a biphasic prospective population-based study during 2014/2015 (1st study) and 2015/2016 (2nd study)^45,46^. Briefly, 65–80-year-old participants (*n* = 34 1st study, *n* = 200 2nd study) were recruited from the general population of Hannover, Germany. The recruitment was performed by taking two independent non-overlapping random samples from the population registry office, so that no participant was included in both studies. We pursued this strategy to be able to confirm the findings from the first study by the results obtained with a completely independent demographically comparable second cohort. All participants provided written informed consent that study data can be used for scientific purposes. Participants received an expense allowance of 25€ covering e.g., public transportation to the study site. The representativeness of these random population-based samples was verified by a non-participant survey^101^. Participants were subjected to a detailed medical interview and physical exam and then vaccinated with Fluad. Blood samples were taken before vaccination (i.e., day 0) and day 1 or 3, day 6 or 7, day 21, and day 70 post-vaccination. Whole blood (PAXgene® tubes for RNA stabilization), plasma, serum, and peripheral blood mononuclear cells (PBMCs; isolated via Ficoll separation for the 1st study or CPT tubes for 2nd study from whole blood) were cryopreserved and stored until further use. Sample numbers for each assay are given in Table S2.

### Antigens and viral preparations

According to the formulation of the Fluad vaccine in the respective seasons, the following antigens or infectious viral particles were used for the hemagglutination inhibition (HAI) assay, the microneutralization (MN) assay, and the antigen-specific stimulation of PBMCs for the flow cytometric analysis: H1N1 A/California/7/09 NYMC-X181 (both seasons), H3N2 A/Texas/50/2012 NYMCX-223 (season 2014/15), H3N2 A/Switzerland/9715293/2013 NIB88 (season 2015/16), B/Massachusetts/02/2012 NYMCBX-51B (season 2014/15), and B/Brisbane/9/2014 (season 2015/16). Infectious virus preparations (NIBSC, UK) were propagated in the chorioallantoic cavity of 10-day-old embryonated eggs for 48 h at 37 °C (H1N1 and H3N1) or 35 °C (B strain)^102^.

### HAI assay to classify vaccine responders and non-responders

To determine HAI titers, serum samples were treated with receptor-destroying enzyme (RDE) at a ratio of 1:4 (1 volume serum and 4 volumes of RDE). Samples from day 0 (before vaccination) and day 21 post-vaccination were used. In the only two cases where no bio sample was available for day 21, serum from 70 days post-vaccination was used. Samples were kept at 37 °C overnight and the reaction was stopped by 30 min incubation at 56 °C. Samples were mixed with the same volume of 0.9% NaCl and frozen at −20 °C until further use. For the HAI assay, serial dilutions of the serum samples were mixed with standardized concentrations of the respective vaccine antigen and incubated with turkey-derived red blood cells. The HAI titer was defined as the highest serum dilution that still resulted in inhibition of hemagglutination. According to the European Medicines Agency (EMA), a post-vaccination titer of ≥40 for vaccinees with a pre-vaccination titer <10 or a ≥ 4-fold increased post-vaccination titer in vaccinees with a pre-vaccination titer ≥10 was considered as positive responsiveness to vaccination and a correlate of protection^103^. We defined triple responders and non-responders as those showing a sero-conversion to all three vaccine antigens or none, respectively.

### Cytokine profiling using serum samples

The cytokine profiling was performed with serum samples using the Immune Monitoring 65-Plex Human ProcartaPlex™ Panel (Invitrogen™/Thermo Fisher Scientific) according to the manufacturer’s protocol. Briefly, serum samples and controls were incubated with the magnetic beads (65 populations with binding sites for the 65 analytes, respectively). Bound analytes were subsequently detected by sequential incubation with detection antibodies and PE-conjugated streptavidin. Samples were measured on a Luminex™ 200 instrument and downstream analysis was performed using the provided software (ProcartaPlex Analyst 1.0). For the further analysis, analytes which were quantifiable in more than three donors of both groups (*n* = 31) were included. For the presentation of the values detected before vaccination (Fig. 2A), responder values were divided by the mean non-responder values at day 0. For the analysis of post-vaccination results (Fig. 2D), all values were divided by the respective day 0 value in order to assess the vaccine-induced impact and normalize for differences observed before vaccination. Subsequently, responder ratios were divided by the non-responder mean ratios for each considered time point, thereby yielding the factor for the comparison of responders and non-responders.

For the pathway integration and visualization, logarithmic (log2) values of the ratio to the respective day 0 value were analyzed using the Pathview online tool^104,105^. For calculating the log2 values, 0 was set to 1 in order to facilitate the conversion. Analyte information was translated into Entrez gene IDs. Responder values were treated as controls and non-responder values as samples for performing an unpaired comparison. The pathway selection was set to “auto” giving a pathway selection based on GAGE for statistical selection^106^.

### Antigen re-stimulation and flow cytometry analysis

Cryopreserved PBMCs were thawed, rested for 2 h, and co-stimulated with purified anti-human CD28 and CD49d at a concentration of 10 µg/ml. For antigen re-stimulation, cells were stimulated with a mixture of the individual monovalent vaccine antigens for the respective season at a concentration of 2.5 µg/each antigen. After 4 h of incubation, brefeldin A and monensin (5 µg/ml) were added to the cells to prevent the secretion of cytokines and receptor internalization and incubated overnight. Cells were stained for flow cytometric analysis using the following antibodies: CD4 (PE-Cy7, L200, Cat.-No. 560909, dilution 1:200), CD19 (APC-Cy7, SJ25C1, Cat.-No. 560177, dilution 1:100), CD14 (APC-Cy7, MΦP9, Cat.-No. 560180, dilution 1:100), CD45RA (FITC, HI100, Cat.-No. 555488, dilution 1:100) and CD57 (PE, NK-1, Cat.-No. 560844, dilution 1:100) from BD (New Jersey, USA); CD4 (BV510, OKT4, Cat.-No. 317444, dilution 1:100), CD8 (BV650, RPA-T8/A700, SK1, Cat.-No. 344730, dilution 1:200), CCR7 (BV785, G043H7, Cat.-No. 353230, dilution 1:100), CD28 (PE-Cy5, CD28.2, Cat.-No. 302910, dilution 1:100), CD24 (BV605, ML5,Cat.-No. 311124, dilution 1:50), IFNγ (A700, 4S.B3, Cat.-No. 502520, dilution 1:200), TNFα (BV605, Mab11, Cat.-No. 502936, dilution 1:200), IL-2 (PB, MQ1-17H12, Cat.-No. 551383, dilution 1:100), IL-10 (PE-Dazzle594, JES3-19F1, Cat.-No. 506812, dilution 1:200), CD127 (BV605, A019D5, Cat.-No. 351334, dilution 1:50 / BV711, A019D5, Cat.-No. 351328, dilution 1:200), CD25 (BV650, BC96, Cat.-No. 302634, dilution 1:50), ICOS(BV421, C398.4A, Cat.-No. 313524, dilution 1:100), CXCR5 (PE-Cy7, J252D4, Cat.-No. 356924, dilution 1:300) and FOXP3 (FITC, 206D, Cat.-No. 320112, dilution 1:100) from BioLegend (San Diego, USA); FOXP3 (AF647, 206D, Cat.-No. 320113, dilution 1:100) from eBioscience/ ThermoFisher Scientific (Massachusetts, USA); and CD3 (BUV395, SK7, Cat.-No. 564001, dilution 1:200), CD19 (A700, HIB19, Cat.-No. 557921, dilution 1:200), CD27 (PerCP-Cy5.5, M-T271, Cat.-No. 560612, dilution 1:100), CD38 (PE-Cy7, HB7, Cat.-No. 335825, dilution 1:100) and CD20 (PE-Cy5, 2H7, Cat.-No. 555624, dilution 1:100) from BD (Franklin Lakes, New Jersey, USA). For surface staining, cells were incubated with the antibody mixture prepared in PBS for 30 min at 4 °C in the dark. For intracellular and intranuclear staining, cells were permeabilized using the Cytofix/Cytoperm kit (BD) or the FOXP3 staining kit (eBioscience), respectively, according to the manufacturer’s instructions. Antibodies were diluted in the respective wash buffer. The samples were acquired at a BD Fortessa flow cytometer using the Diva software and analyzed using FlowJo. Missing values in the shown data sets arose due to sample availability (e.g. sampling time points, amount of frozen PBMCs) or samples/values were excluded due to processing or measurement inconsistencies. Missing single values are indicated in the Source Data file. For the Stochastic neighbor embedding (t-distributed) analysis (tSNE), compensated samples were randomly down-sampled with regard to the number of acquired cells. This was accomplished by using the respective plug-in to reduce the event rate to 3000 events per individual sample per time point. This was done in order to exclude any bias resulting from the inclusion of different cell numbers. The reduced samples were labeled, concatenated and the tSNE analysis was performed using the respective plug-in. The resulting data were plotted with intensities for the depicted markers. The statistical analysis was performed using GraphPad Prism (version 6.0 and 8.4.3 for Windows, GraphPad Software, La Jolla, California USA, www.graphpad.com) applying student’s t-test and two-way ANOVA in combination with the uncorrected Fisher’s LSD test or Mixed-effects analysis in cases with missing values. The uncorrected Fisher’s LSD test is a follow-up test of an ANOVA and represents a set of individual *t* tests, thus not correcting for multiple comparisons.

### Analysis of the T_REG_‐specific demethylated region (TSDR)

For the sorting of CD4^+^ T cells, PBMCs were labeled with anti-CD3 (AF488, UCHT1, Cat.-No.300415, dilution 1:200) and anti-CD4 (AF647, RPA-T4, Cat.-No. 300520, dilution 1:200) conjugates (BioLegend). Dead cells were excluded using the LIVE/DEAD™ Fixable Near-IR Dead Cell Stain Kit (ThermoFisher Scientific, Waltham, USA). Cells were sorted as living CD3^+^CD4^+^ cells. DNA was subsequently extracted from sorted cells using the DNeasy blood and tissue kit (Qiagen, Hilden, Germany), according to the manufacturer’s instructions. Afterward, the extracted DNA was concentrated using DNA Clean & Concentrator™−10 (Zymo Research, Irvine, USA). Bisulfite conversion was performed as described earlier^107^. Methylation‐specific real‐time PCR was performed as recently described^108,109^, using a minimum of 60 ng bisulfite‐treated genomic DNA. Results are reported as percentage of T cells with demethylated TSDR.

### Bulk transcriptome analysis

For the bulk transcriptome analysis, whole blood samples frozen in PAXgene™ tubes (BD) were used. The “PAXgene Blood miRNA Kit” (Qiagen) was used for the purification of miRNA and total RNA. The quality and integrity of the resulting total RNA were controlled on Agilent Technologies 2100 Bioanalyzer (Agilent Technologies; Waldbronn, Germany). 100 ng total RNA was depleted for rRNA and globin by using the QIAseq® FastSelectTM RNA Removal Kit (Qiagen). The RNA Sequencing library was prepared with NEBNext® Ultra™ II Directional RNA Library Prep Kit for Illumina® (New England Biolabs). The libraries were sequenced on Illumina NovaSeq 6000 using NovaSeq 6000 S2 Reagent Kit (100 cycles, paired-ended run) with an average of 50 × 10^6^ reads per RNA sample.

### Single-cell RNA sequencing

For the single cell analysis, cryopreserved PBMCs derived from three responders and three non-responders taken before and 7 days post-vaccination were thawed and re-stimulated with the antigen mixture as described above for the flow cytometry approach. Subsequently, CD45-expressing cells were sorted and loaded into a well on a 10× Chromium Single Cell instrument (10x Genomics). The cDNA libraries were constructed using the 10× Chromium™ Single cell 5′ Library Kit according to the manufacturer’s original protocol. Libraries were sequenced on an Illumina NovaSeq 6000 2 × 50 paired-end kits using the following read length: 26 bp Read1 for cell barcode and a unique molecular identifier (UMI), 8 bp I7 index for sample index and 89 bp Read2 for transcript. Cell Ranger 3.0.1 (http://10xgenomics.com) was used to process the Chromium single cell 5′ RNA-seq output. First, “cellranger mkfastq” demultiplexed the sequencing samples based on the 8 bp sample index read to generate fastq files for the Read1 and Read2, followed by extraction of 16 bp cell barcode and 10 bp UMI. Second, “cellranger count” aligned the Read2 to the human reference genome (GRCh38) using STAR. Then, aligned reads were used to generate a data matrix only when they had valid barcodes and UMI and when they mapped to exons without PCR duplicates. Valid cell barcodes were defined based on UMI distribution.

Data normalization and clustering of single-cell study: UMI count matrices were imported to R/Seurat package v3.1 for downstream analyses. For quality control, we excluded genes that were expressed in less than three cells. We further excluded cells with more than 15% mitochondrial reads, less than 100 or more than 3000 expressed genes and more than 10,000 detected transcripts. LogNormalization in Seurat package was applied before downstream analysis. The original gene counts for each cell were normalized by total UMI counts, multiplied by 10,000 (TP10k) and the log-transformed by log10 (TP10k + 1). Subsequently, the data was scaled, centered, and regressed against the number of detected transcripts per cell to correct for heterogeneity associated with differences in sequencing depth. After correction, principal component analysis (PCA) was performed based on the 2000 most variable features identified using the vst method implemented in Seurat. The cells were then clustered using the Louvain algorithm based on the first 20 “PCA” dimensions with a resolution of 0.4. For two-dimensional data visualization, we performed UMAP also based on the first 20 “pca” dimensions. Cell population annotation was based on the respective clustering results combined with data-driven classification from SingleR and expression of known marker genes^110^.

Differentially expressed (DE) genes in scRNA-seq: DE tests were estimated using FindMarkers/FindAllMarkers functions in Seurat v3.1 with the Wilcoxon rank-sum test. For each comparison, unless specifically described, genes that were expressed at least 10% in the tested group and had Bonferroni corrected *P* values ≤0.05 were regarded as significantly differentially expressed (DE) genes. Cluster or sub-group marker genes were identified by applying DE tests for upregulated genes between cells in one cluster/sub-group to all other clusters in the tested dataset.

Quantification of the percentages of cell populations in the PBMC scRNA-seq data: To compare shifts in the PBMC compartment of responder and non-responder volunteers, the percentages of the cellular subsets, as identified by clustering and cluster annotation explained above for the two-time points of scRNA-seq datasets (D0 and D7), of the total number of PBMC in each dataset were quantified per sample and visualized together in boxplots. To determine the statistical significance of differences in cell proportions between the responders and non-responders, a Dirichlet regression model (R/DirichletReg package) was used accounting for compositional dependencies (e.g. non-independence)^111,112^. Considering the proportions of all cell types sum to 1 in each sample, we used the Dirichlet-multinomial regression model to test for the differences in proportion of each cell type between sample type (responder vs. non-responder) or sample time (day 0, day 7), while accounting proportions of all other cell types as dependencies. This analysis was performed based on the scripts provided in ref. 112. The *p* values were corrected for multiple testing using the Benjamini–Hochberg procedure.

### Mathematical modeling

The expansion rates were modeled using the absolute cell numbers of different cell types in the group of responders and non-responders at day 0, day 3, and/or day 6. The MATLAB 2016a function (fit) was employed to fit linearly (*y* = *mx* + *b*) the first increase observed between day 0 and 3 in each cell type, allowing to record the slope for each vaccinee. Note that this term is broadly defined as “expansion”. However, this term can be a combination of different events happening in the blood compartment after vaccination, e.g. proliferation, migration, and differentiation. The area under the receiver operating characteristic (ROC) curve (AUC)^113–115^ is computed. Here, the higher the AUC, the better the model is at distinguishing between responders and non-responders with a value of 1 describing a model with an outstanding discrimination. Two-tailed tests were used to assess the significance between-group differences: the two-sample t-test (MATLAB function ttest2) and the Wilcoxon rank sum test (MATLAB function ranksum).

### Reporting summary

Further information on research design is available in the Nature Portfolio Reporting Summary linked to this article.

## Supplementary information

Supplementary Information

Reporting Summary

## Data Availability

Source data are provided with this paper. RNA sequencing data sets that support the findings of this study are available from https://www.ncbi.nlm.nih.gov/geo/query/acc.cgi?acc=GSE211368 (bulk RNA sequencing) and https://www.ncbi.nlm.nih.gov/geo/query/acc.cgi?acc=GSE211560 (scRNA sequencing). The microbiome sequencing data set is available from https://www.ebi.ac.uk/ena/browser/view/PRJEB56539. Source data are provided with this paper.

## Source data

Source Data
