# Supplementary material for: Distinct immunological and molecular signatures underpinning influenza vaccine responsiveness in the elderly
Source: Nat Commun. 2022 Nov 12;13:6894. doi: 10.1038/s41467-022-34487-z (PMC9653450; doi:10.1038/s41467-022-34487-z)
Supplement: Supplementary file 1 — Supplementary Information [file 41467_2022_34487_MOESM1_ESM.pdf]

Distinct immunological and molecular signatures underpinning influenza vaccine responsiveness in the elderly

Riese, Trittel et al.

Supplementary Information

Supplementary Figure 1

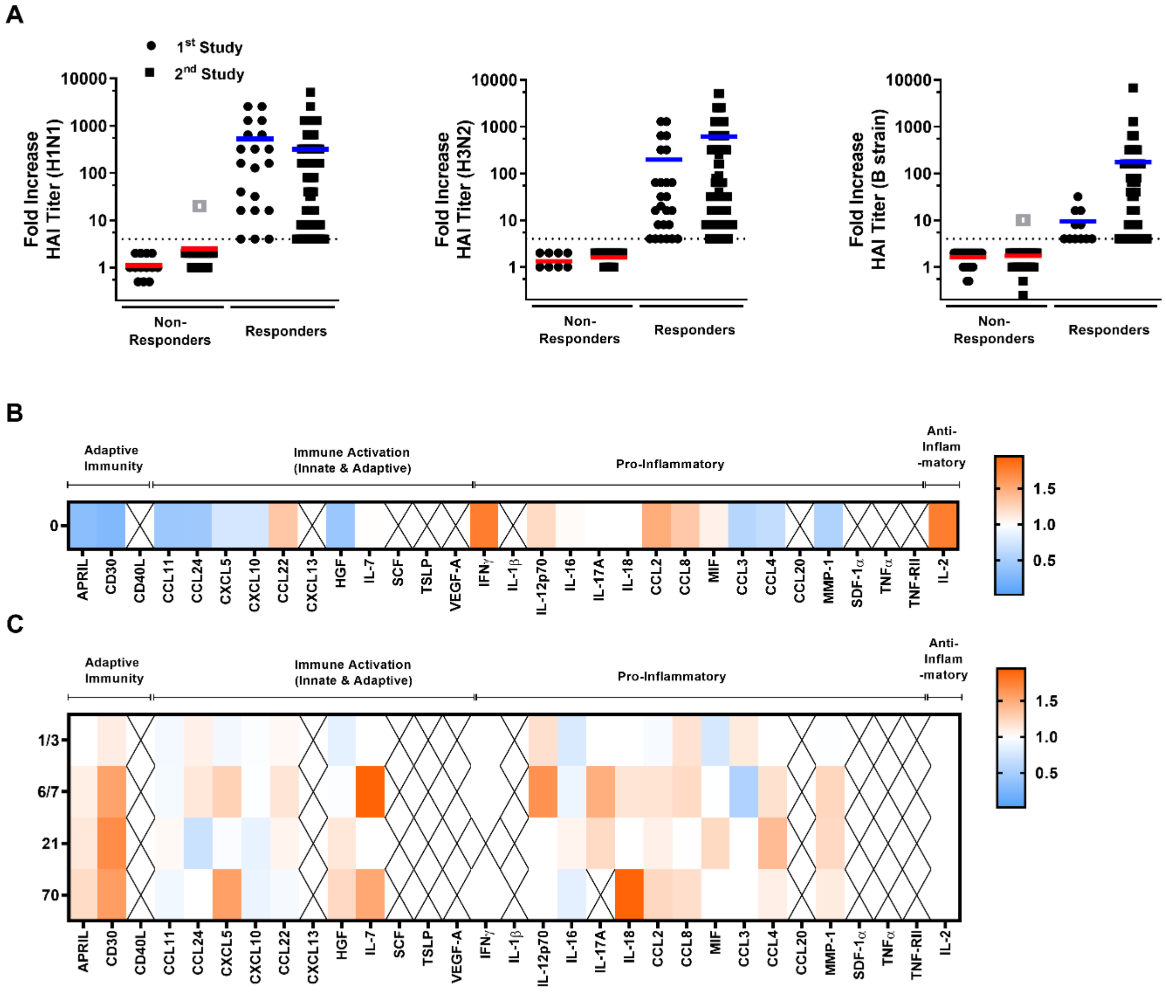

Supplementary Figure 1: Response rates and cytokine profile of vaccinees. (A) Serum samples of all study participants (1st study filled circles, 2nd study filled squares) were subjected to HAI testing against all the vaccine antigens (n=234 biologically independent samples). (B) Heat map showing the ratio of cytokine concentrations detected before vaccination in responders vs. non-responders. Red indicates values with a higher expression in responders, blue indicates values with a higher expression in non-responders (1st study, n=13 biologically independent samples). (C) Heat map depicting the ratio of vaccine-induced serum factors (fold increase over day 0) for triple vaccine responders as compared to non-responders (orange represents values that show a higher response in responders as compared to non-responders, blue indicates a lower response in responders as compared to non-responders, 1st study, n=13 biologically independent samples). Source data are provided as a Source Data file.

Supplementary Figure 2

A

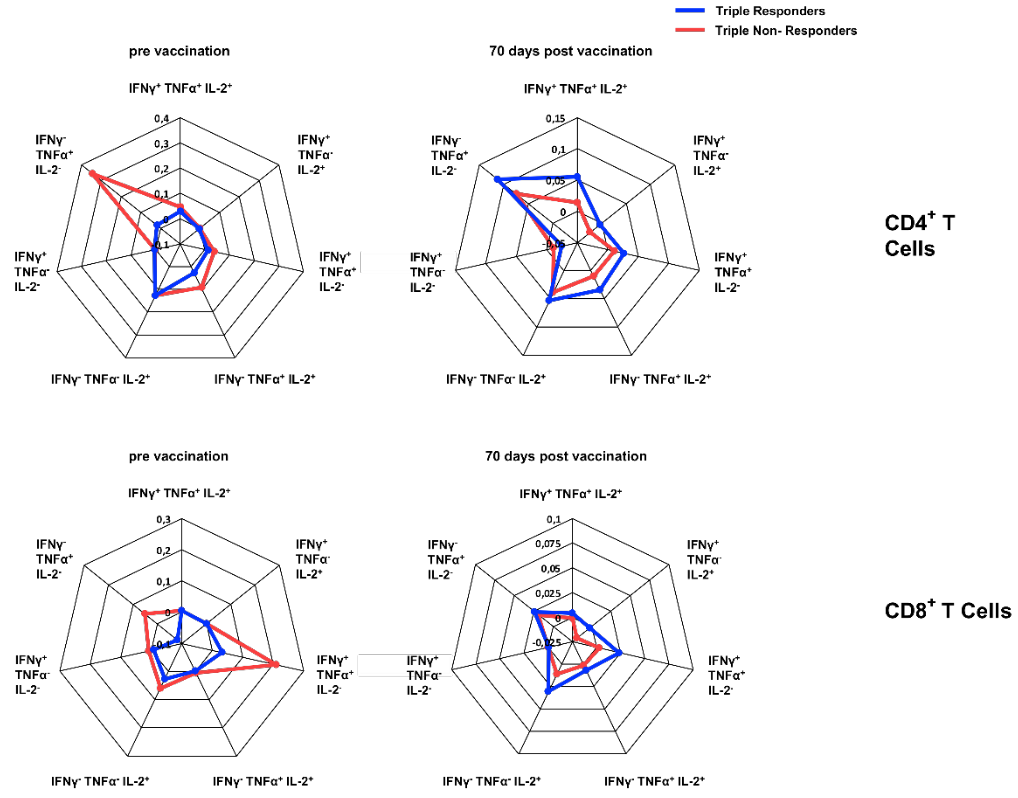

B

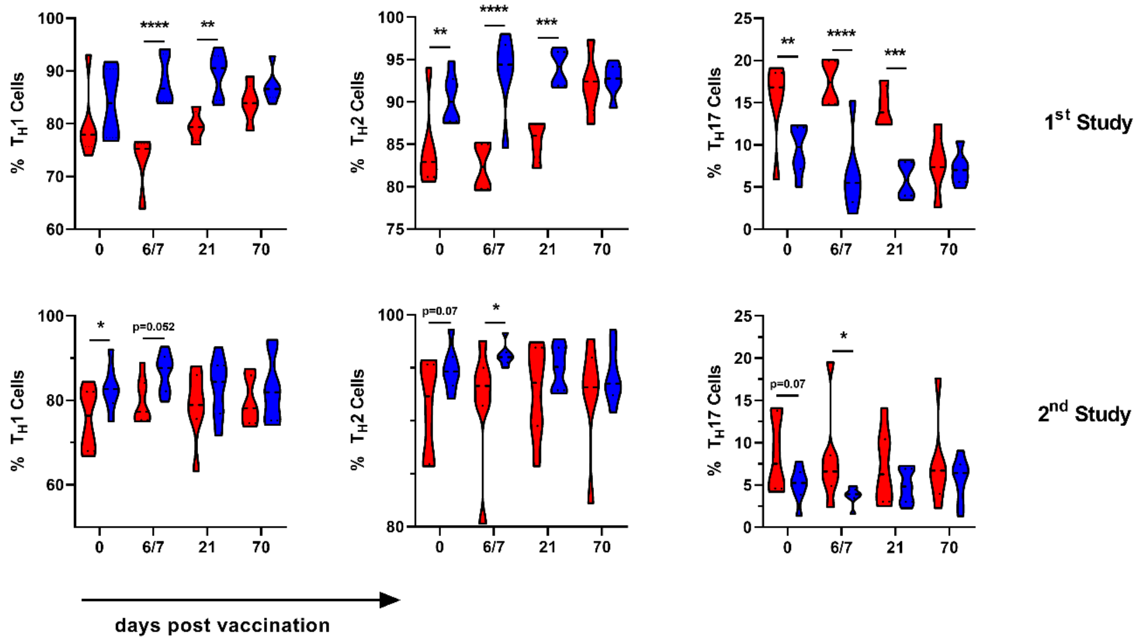

C

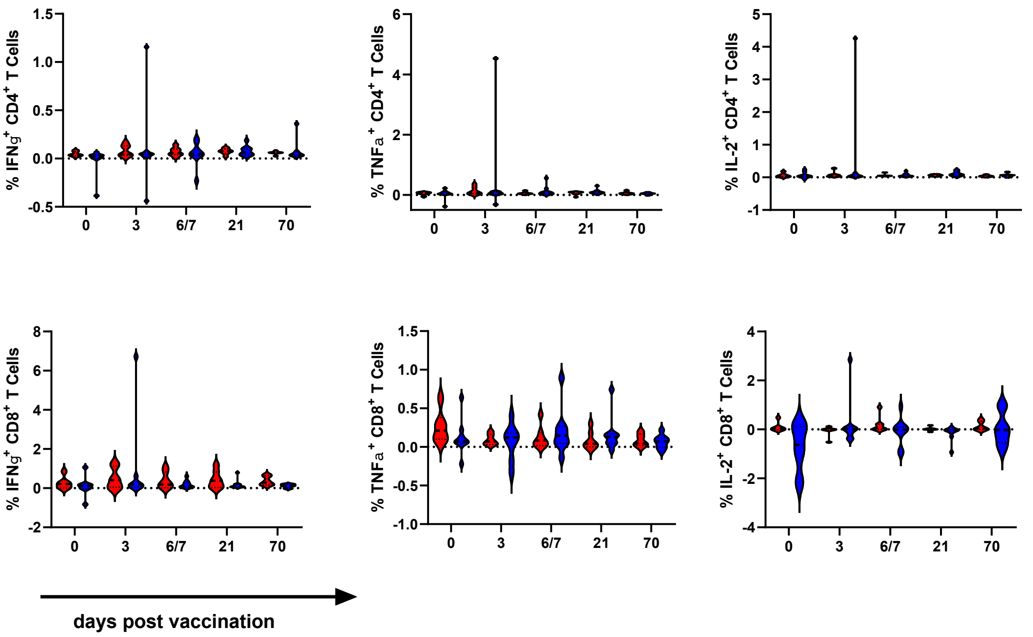
% IFNg+ CD4+ T Cells
% TNFa + CD4+ T Cells
% IL-2+ CD4+ T Cells
% IFNg+ CD8+ T Cells
% TNFa + CD8+ T Cells
% IL-2+ CD8+ T Cells
0
3
6/7
21
70
days post vaccination

D

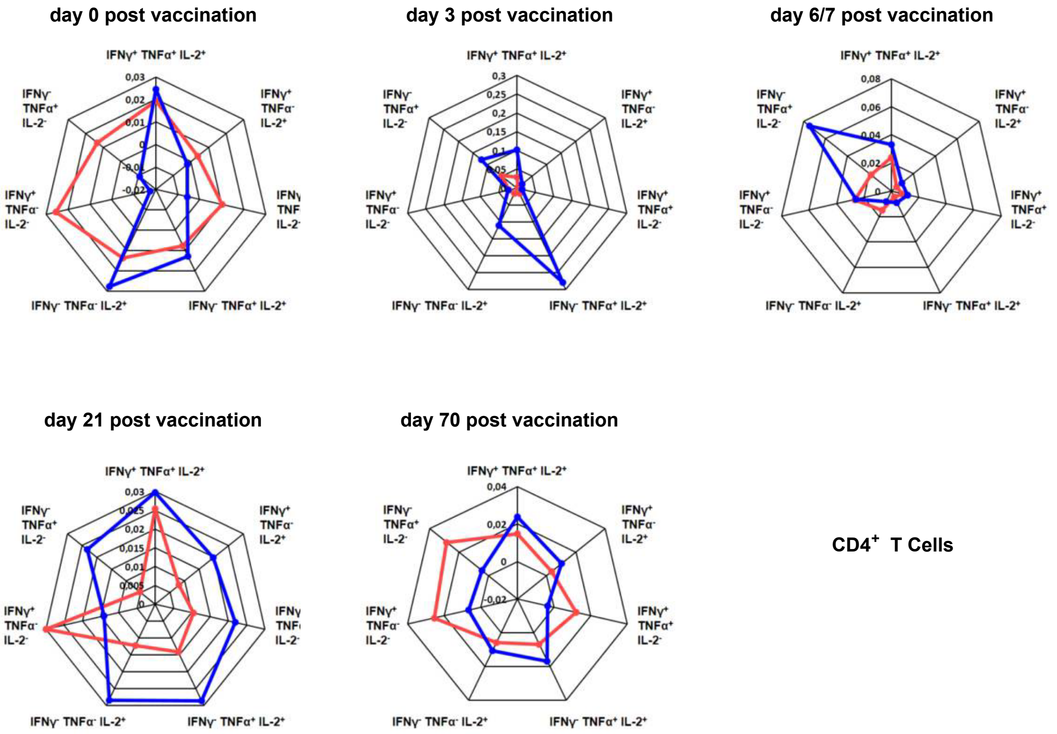
day 0 post vaccination
day 3 post vaccination
day 6/7 post vaccination
day 21 post vaccination
day 70 post vaccination
IFNγ+ TNFα+ IL-2+
IFNγ+ TNFα- IL-2+
IFNγ+ TNFα+ IL-2-
IFNγ- TNFα+ IL-2+
IFNγ- TNFα- IL-2+
IFNγ+ TNFα- IL-2-
IFNγ- TNFα+ IL-2-
CD4+ T Cells

E

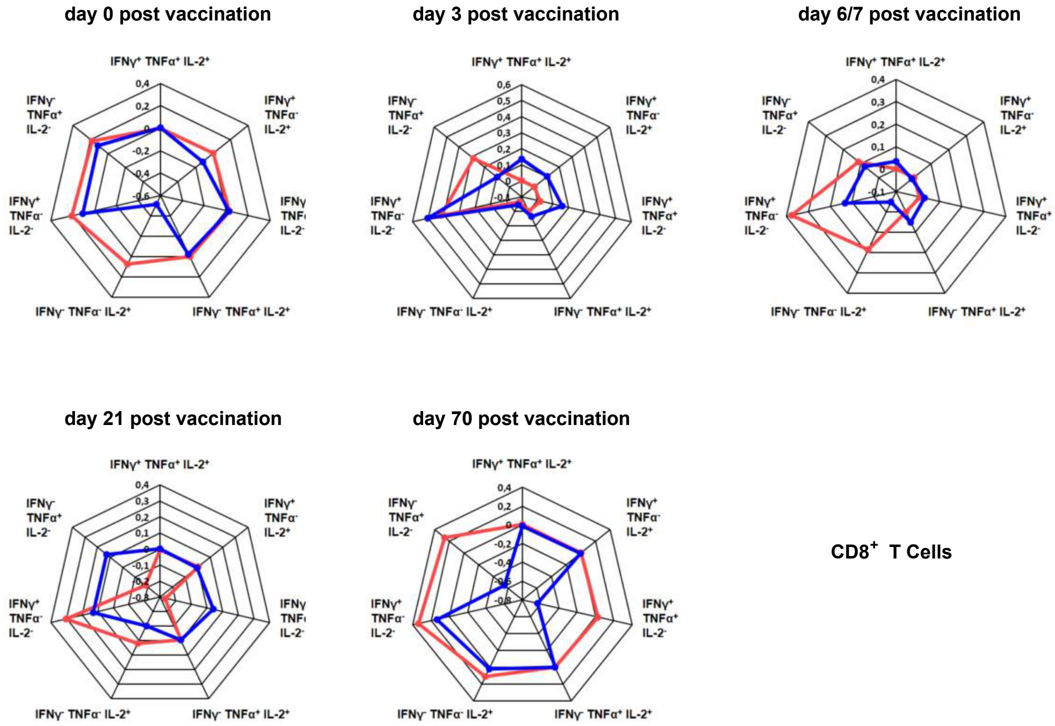

F

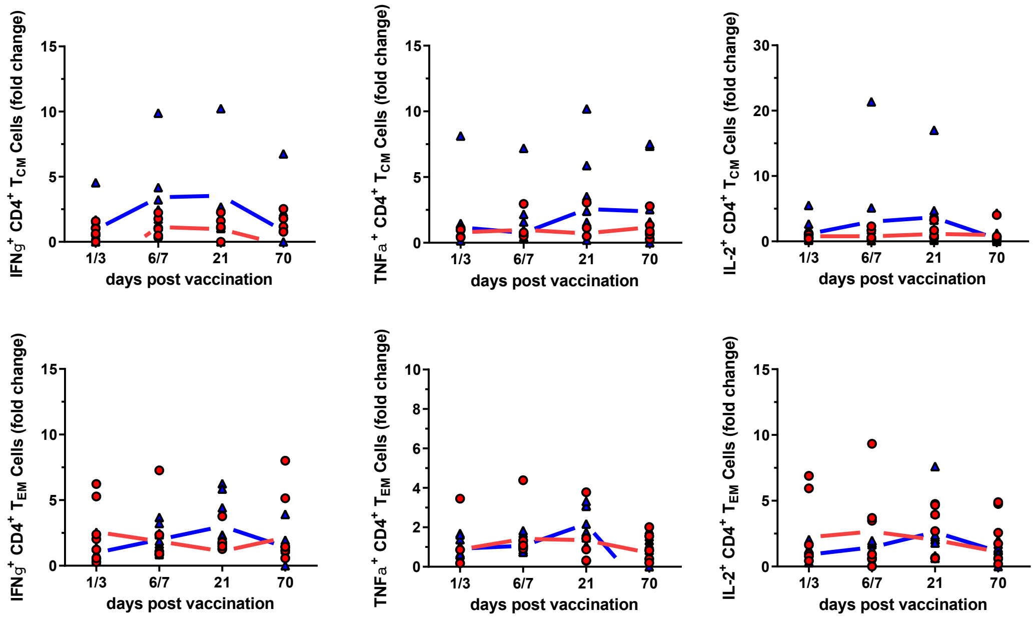

Figure S2: T_H subset distribution in triple vaccine responders and non-responders. Cryopreserved PBMCs isolated from triple responders and non-responders were left unstimulated or re-stimulated with the vaccine antigens overnight, stained for surface antigens and intracellular cytokine production and analyzed by flow cytometry. (A) Frequencies of $IFN\gamma^+$, $TNF\alpha^+$ and/or $IL\text{-}2^+$ $CD4^+$ and $CD8^+$ T cells before and 21 days post vaccination depicted as spider plots (1st study, n=6 and n=6 non-responders and responders, respectively, biologically independent samples). (B) T_H subsets were defined as: T_H1 ($CD4^+CXCR3^+CCR6^-$, 1st study day 6/7 triple non-responders vs. responders $p < 0.0001$, 1st study day 21 triple non-responders vs. responders $p=0.0040$, 2nd study day 0 triple non-responders vs. responders $p=0.0439$), T_H2 ($CD4^+CXCR3^-CCR6^-$, 1st study day 0 triple non-responders vs. responders $p=0.0048$, 1st study day 6/7 triple non-responders vs. responders $p <0.0001$, 1st study day 21 triple non-responders vs. responders $p=0.0004$, 2nd study day 6/7 triple non-responders vs. responders $p=0.0261$) and T_H17 ($CD4^+CXCR3^-CCR6^+$, 1st study day 0 triple non-responders vs.

responders p=0.0048, 1st study day 6/7 triple non-responders vs. responders p <0.0001, 1st study day 21 triple non-responders vs. responders p=0.0004, 2nd study day 6/7 triple non-responders vs. responders p=0.0260) cells (unstimulated samples) (1st study, n=6 and n=6 non-responders and responders, respectively, 2nd study, n=7 non-responders, n=8 responders, biologically independent samples). Violin plots show the mean and the quartiles as dashed and dotted lines, respectively. The shape indicated the data distribution. (C) Frequencies of IFNγ$^+$, TNFα$^+$ and IL-2$^+$ CD4$^+$ T cells and CD8$^+$ T cells. Violin plots represent data subtracted for background functionality (2nd study, n= 7 non-responders, n=9 responders, biologically independent samples). (D) Frequencies of multifunctional (IFNγ$^+$, TNFα$^+$ and/or IL-2$^+$) CD4$^+$ T cells and (E) multifunctional CD8+ T cells depicted as spider plots (2nd study, n=8 non-responders, n= 8-9 responders, biologically independent samples). (F) Frequencies of IFNγ$^+$, TNFα$^+$ and IL-2$^+$ central memory CD4$^+$ T cells (CD45RA$^-$CCR7$^+$) and effector memory CD4$^+$ T cells (CD45RA$^-$CCR7$^-$) depicted as scatter plots with connecting lines (2nd study, n=7 non-responders and n=10 responders, biologically independent samples). Lines show the connected median values and symbols indicate single values. Asterisks denote statistical significance as calculated by Two-way ANOVA/ Mixed-effects analysis based on nominal p values (uncorrected Fisher's LSD) comparing triple vaccine responders and non-responders at each time point. Source data are provided as a Source Data file.

Supplementary Figure 3

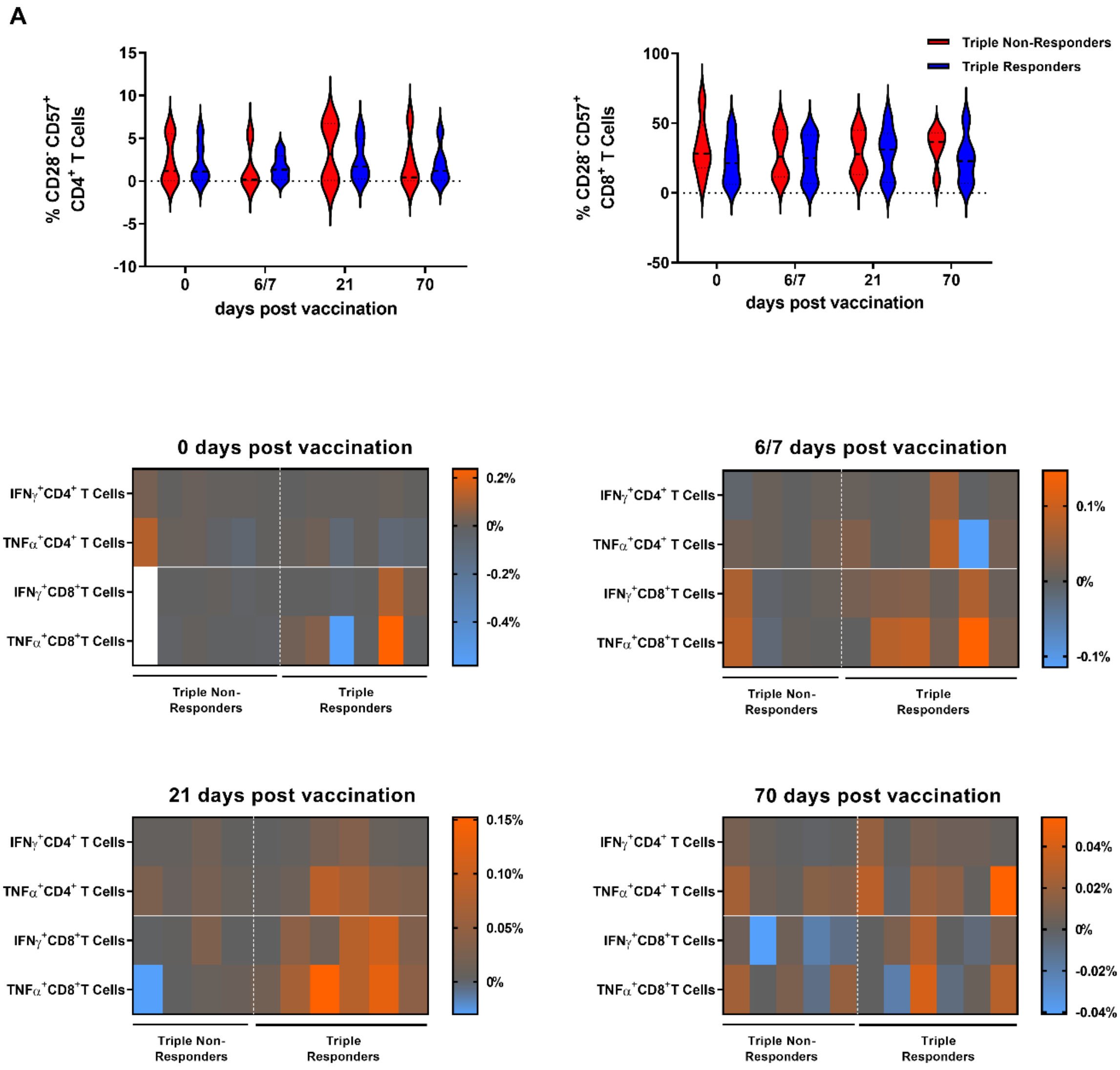

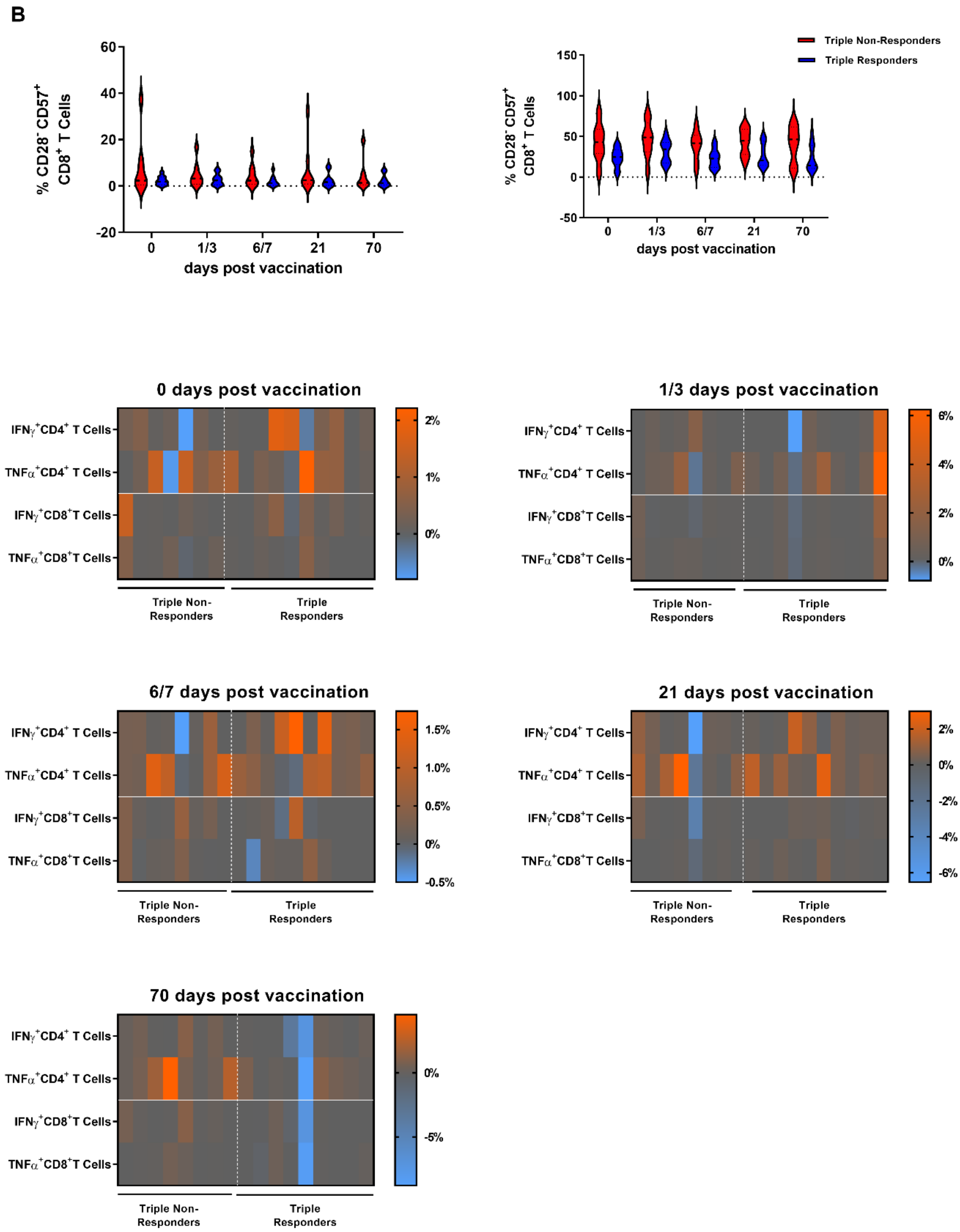

Figure S3: Phenotype and functionality of senescent $CD4^+$ and $CD8^+$ T cells. Cryopreserved PBMCs isolated from triple responders and non-responders were left unstimulated or re-stimulated with the vaccine antigens overnight, stained for surface antigens and intracellular cytokine production and analyzed by flow cytometry. Frequencies of senescent ($CD28^-CD57^+$) $CD4^+$ and $CD8^+$ T cells and cytokine-secreting senescent antigen-specific T cells (subtracted background) at the indicated time points. (A) 1st study (n=6 non-responders and n=6 responders, biologically independent samples) and (B) 2nd study (n=8 non-responders and 10 responders, biologically independent samples). Violin plots show the mean and the quartiles as dashed and dotted lines, respectively. The shape indicated the data distribution. Heat maps show the frequencies of $IFN\gamma^+$ and $TNF\alpha^+$ expressing $CD4^+$ and $CD8^+$ T cells. Source data are provided as a Source Data file.

Supplementary Figure 4

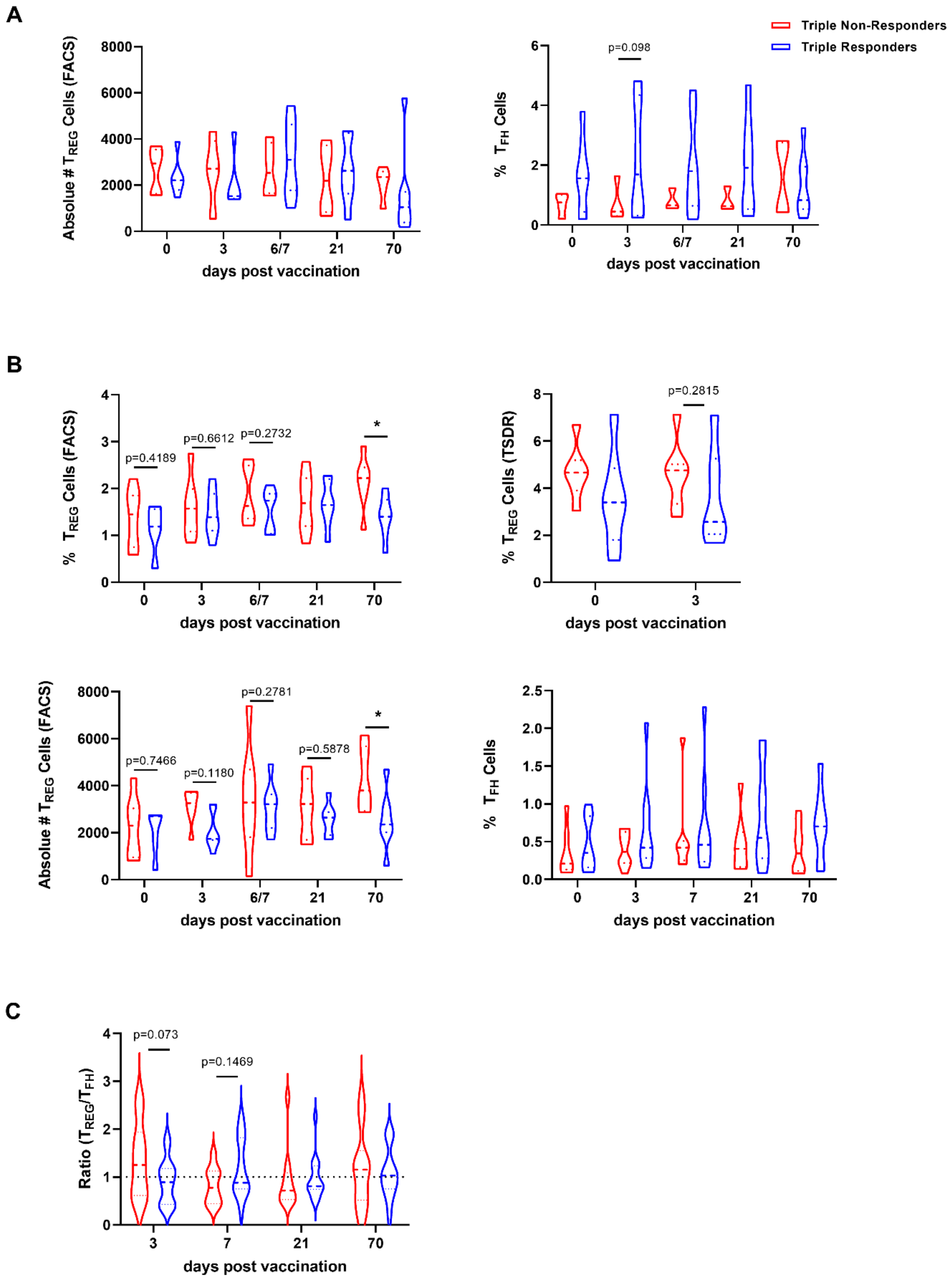

Figure S4: T_{REG} and T_{FH} cells as important factors for vaccine-induced immune responses. Cryopreserved PBMCs isolated from vaccine triple responders and non-responders were stained for surface antigens and intranuclear expression of FOXP3 *ex vivo* and analyzed by flow cytometry. (A) Absolute numbers of T_{REG} cells ($CD4^{+}CD127^{low}CD25^{+}FOXP3^{+}$) and frequencies of T_{FH} cells

($CD4^+CXCR5^+ICOS^+$) assessed by flow cytometry (1st study, n=6 non-responders and n=7 responders, biologically independent samples). (B) Frequencies of T_{REG} cells assessed by flow cytometry (day 70 comparison triple non-responders vs. responders p=0.0134) and TSDR analysis and absolute numbers (day 70 comparison triple non-responders vs. responders p=0.0107) (2nd study, n=8 non-responders and n=9 responders, biologically independent samples) and frequencies of T_{FH} cells ($CD4^+CXCR5^+ICOS^+$) assessed by flow cytometry (2nd study, n=17 biologically independent samples). (C) Mean values of T_{REG} and T_{FH} cell frequencies at the indicated time points post vaccination (1st & 2nd study, n=11 non-responders and n=15 responders). Violin plots show the mean and the quartiles as dashed and dotted lines, respectively. The shape indicated the data distribution. Asterisks denote significant values as calculated by Two-way ANOVA/ Mixed-effects analysis without correction for multiple comparisons (uncorrected Fisher's LSD) comparing triple vaccine responders and non-responders at a given time point. Source data are provided as a Source Data file.

Supplementary Figure 5

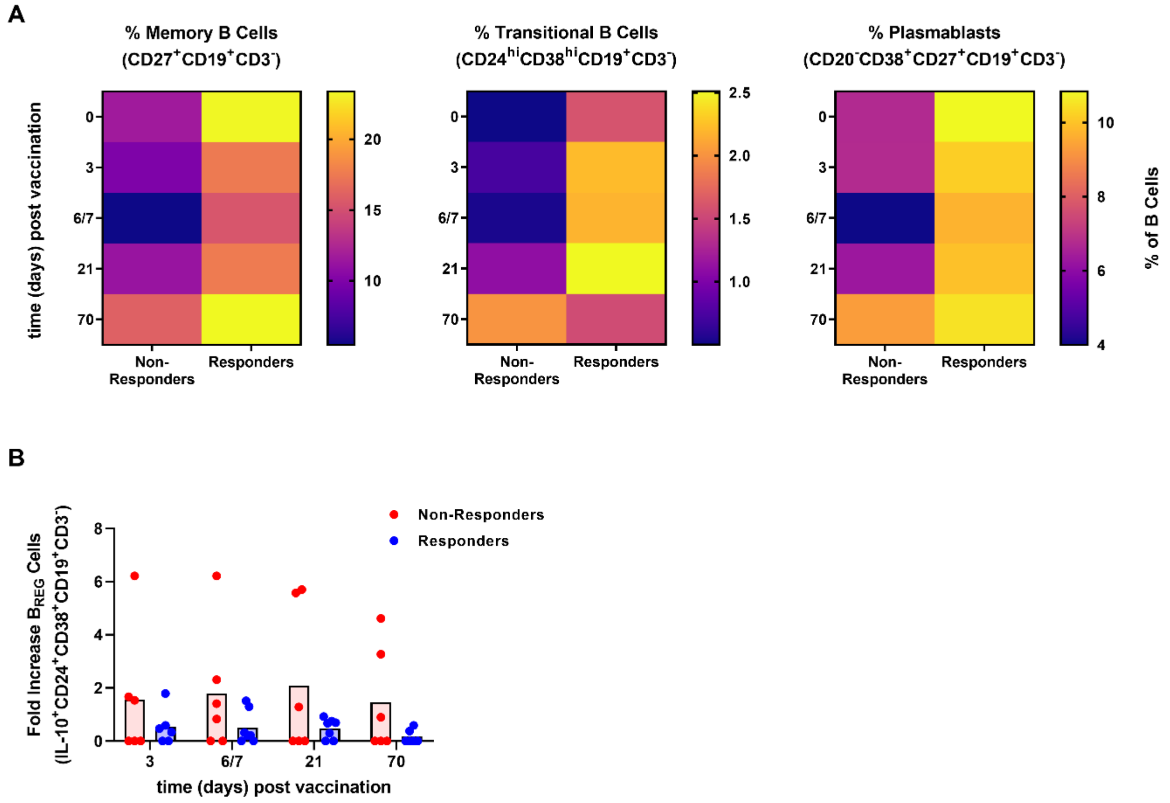

Figure S5: B cell responses in vaccine responders and non-responders. Cryopreserved PBMCs isolated from vaccine triple responders and non-responders were stimulated with the vaccine antigens and stained for surface antigens identifying (A) memory B cells ($CD27^+CD19^+CD3^-$), transitional B cells ($CD24^{hi}CD38^{hi}CD19^+CD3^-$) and plasmablasts ($CD20^-CD38^+CD27^+CD19^+CD3^-$). Heat maps show the means of frequencies (% of $CD19^+CD3^-$ B cells) of re-stimulated samples (1st study, n=5 non-responders and n=7 responders). (B) Fold increase of B_{REG} cells ($IL\text{-}10^+CD24^+CD38^+CD19^+CD3^-$) at the indicated time points as compared to day 0 (1st study, n=6 non-responders and n=7 responders). Columns represent the mean ± SEM of data from re-stimulated samples with individual values depicted as dots. Asterisks denote significant values as calculated by Two-way ANOVA/ Mixed-effects analysis without correction for multiple comparisons (uncorrected Fisher's LSD) comparing triple vaccine responders and non-responders at a given time point. Source data are provided as a Source Data file.

Supplementary Figure 6

A

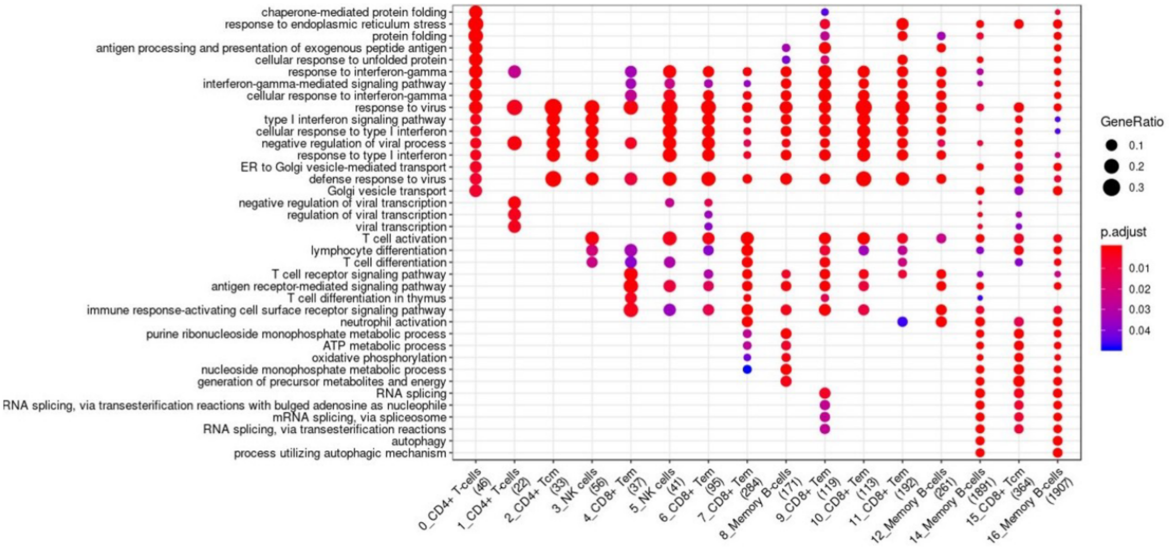

B

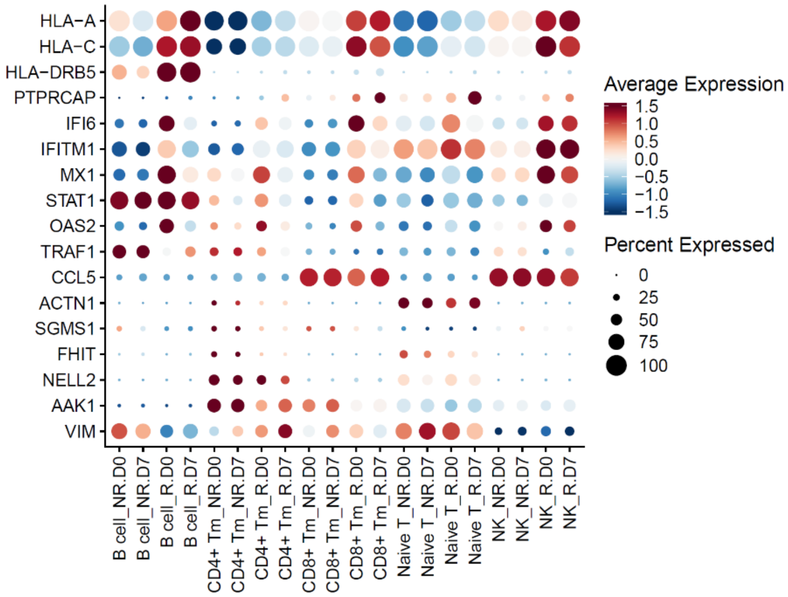

C

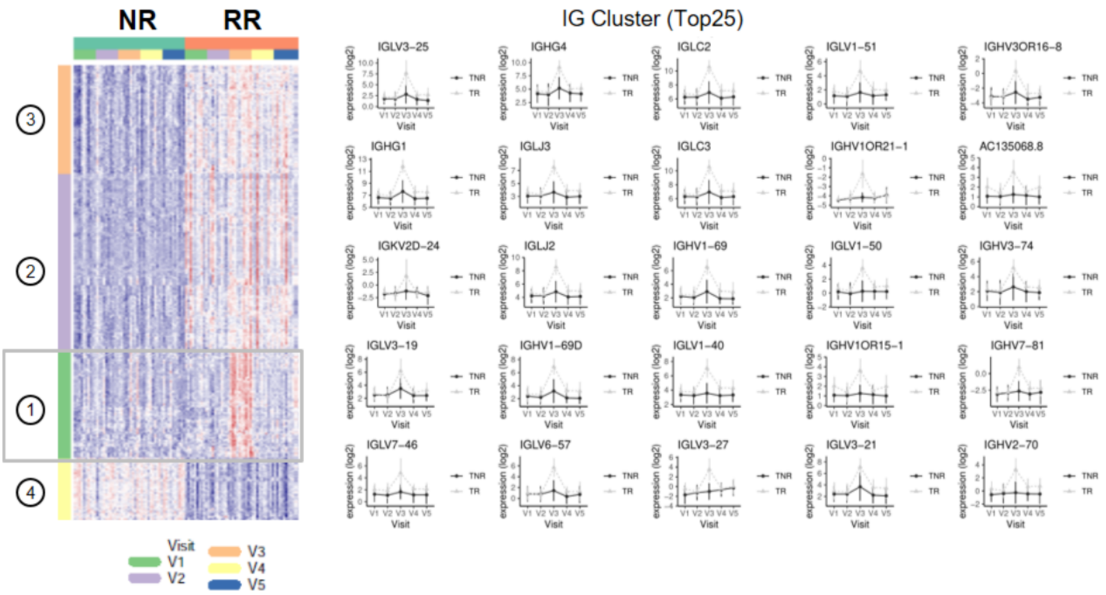

D

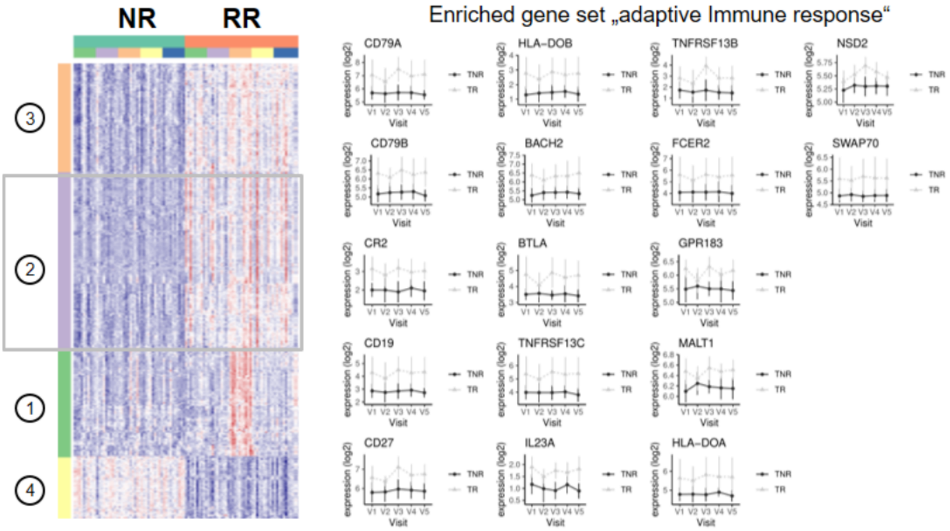

Figure S6: Transcriptome analyses of vaccine responders and non-responders. For the scRNA Seq analysis, cryopreserved PBMCs were re-stimulated with the vaccine antigen mix and subjected to sorting for CD45+ cells prior to the further measurements (n=6; three responders/ three non-responders, before and 7 days post vaccination). (A) Gene expression profiles assigned to cellular pathways comparing responders to non-responders (circles = gene ratios → the bigger the circle, the higher the expression in responders as compared to non-responders, red = low p value = highly significant difference between, blue = high p value = small difference). (B) Genes differentially regulated in responders as compared to non-responders assigned to the identified cell clusters. For the bulk transcriptome analysis, blood samples were cryopreserved in PAXgene® blood RNA tubes and the isolated RNA was applied to a transcriptomic analysis. Identified clusters and related pathways were identified. Gene expression patterns in (C) cluster 1 and (D) cluster 2 (IG = immunoglobulin).

Supplementary Figure 7

A

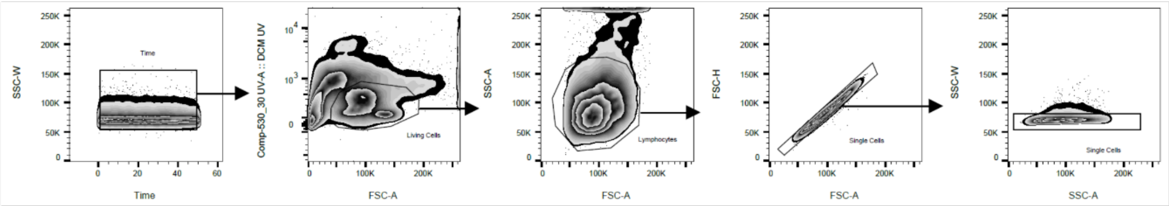

B

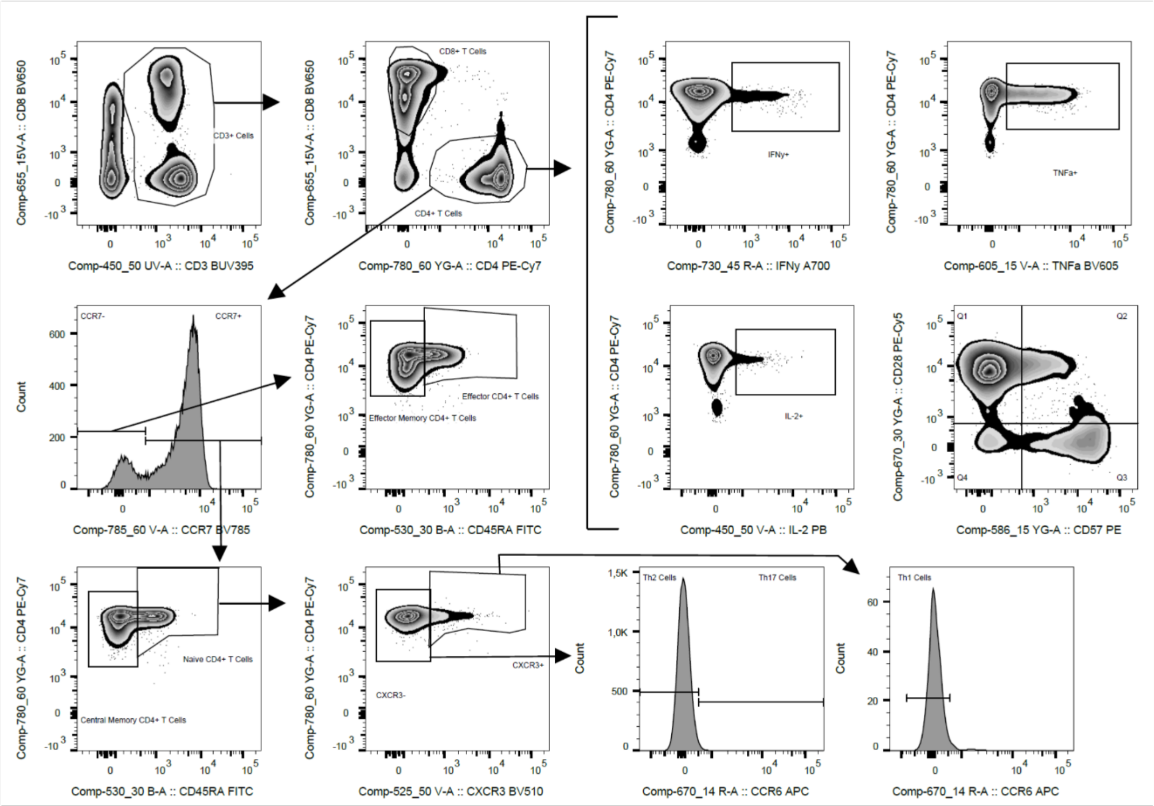

C

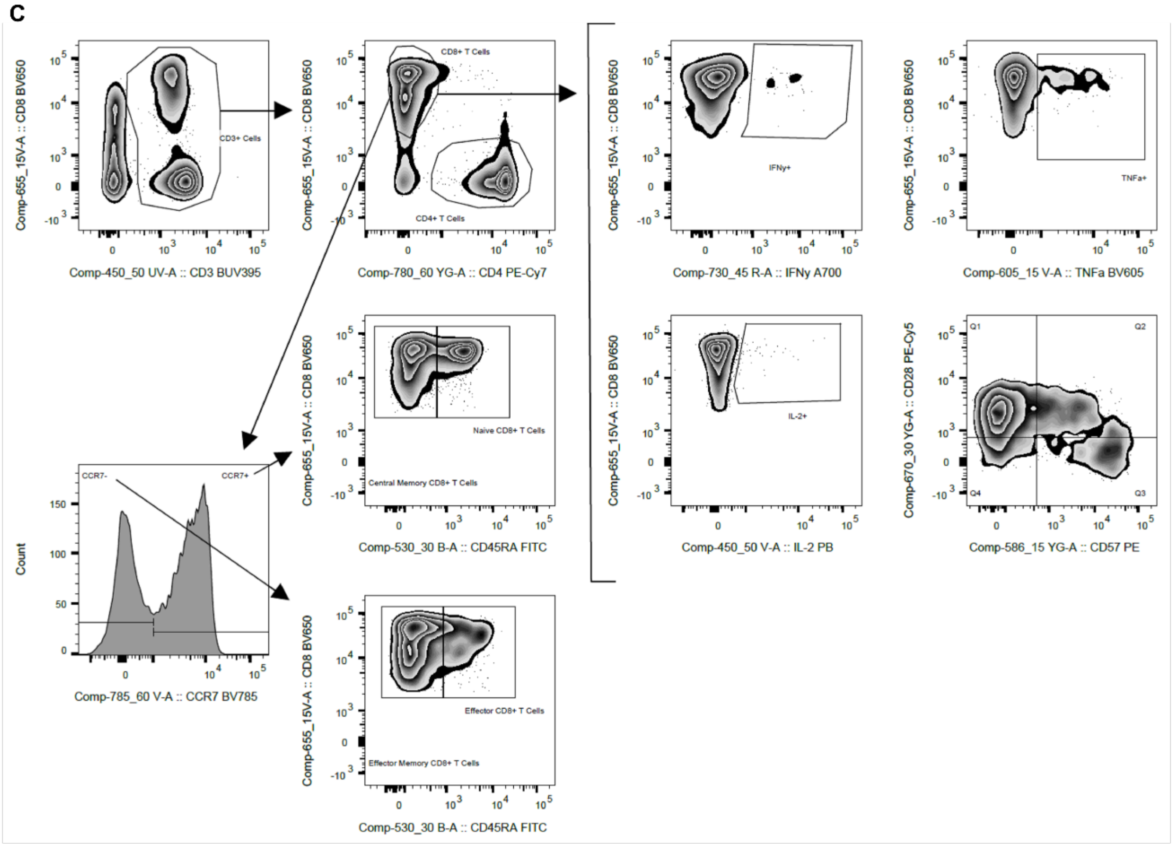

Comp-655_15V-A :: CD8 BV650
Comp-450_50 UV-A :: CD3 BUV395
CD3+ Cells
CD8+ T Cells
CD4+ T Cells
Comp-780_60 YG-A :: CD4 PE-Cy7
IFNy+
Comp-730_45 R-A :: IFNy A700
TNFa+
Comp-605_15 V-A :: TNFa BV605
Naive CD8+ T Cells
Central Memory CD8+ T Cells
Comp-530_30 B-A :: CD45RA FITC
IL-2+
Comp-450_50 V-A :: IL-2 PB
Comp-670_30 YG-A :: CD28 PE-Cy5
Comp-586_15 YG-A :: CD57 PE
Q1
Q2
Q3
Q4
CCR7-
CCR7+
Count
Comp-785_60 V-A :: CCR7 BV785
Effector CD8+ T Cells
Effector Memory CD8+ T Cells

D

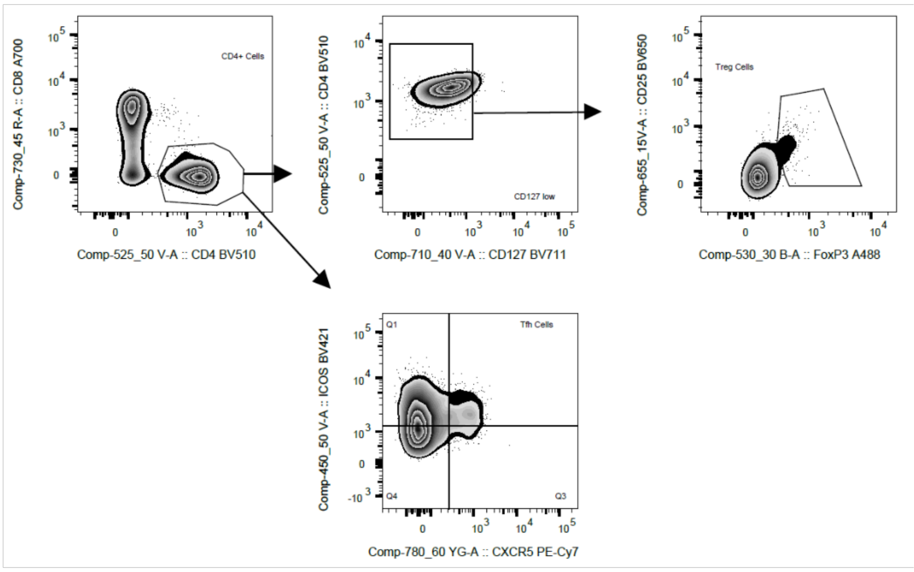

Comp-730_45 R-A :: CD8 A700
CD4+ Cells
Comp-525_50 V-A :: CD4 BV510
CD127 low
Comp-710_40 V-A :: CD127 BV711
Comp-655_15V-A :: CD25 BV650
Treg Cells
Comp-530_30 B-A :: FoxP3 A488
Comp-450_50 V-A :: ICOS BV421
Tfh Cells
Q1
Q3
Q4
Comp-780_60 YG-A :: CXCR5 PE-Cy7

E

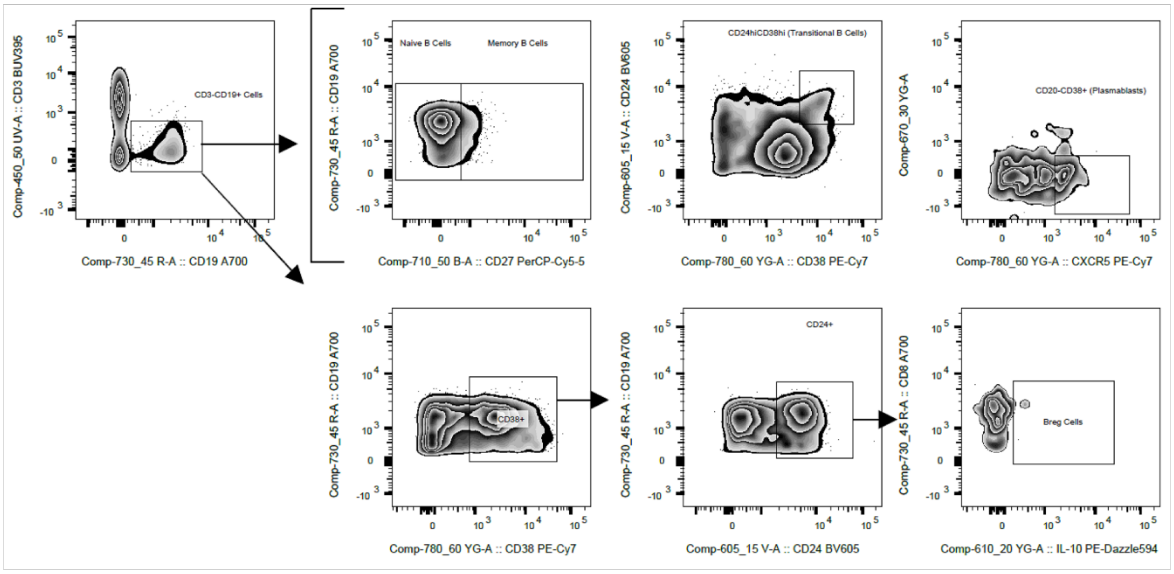

Figure S7: Gating strategies. All FACS raw data was first gated for living single lymphocytes according to (A). Subsequently, cells were gated for (B) $CD4^+$ T cell populations, (C) $CD8^+$ T cell populations, (D) T_{REG} and T_{FH} cells or (E) B cell populations.

Supplementary Figure 8

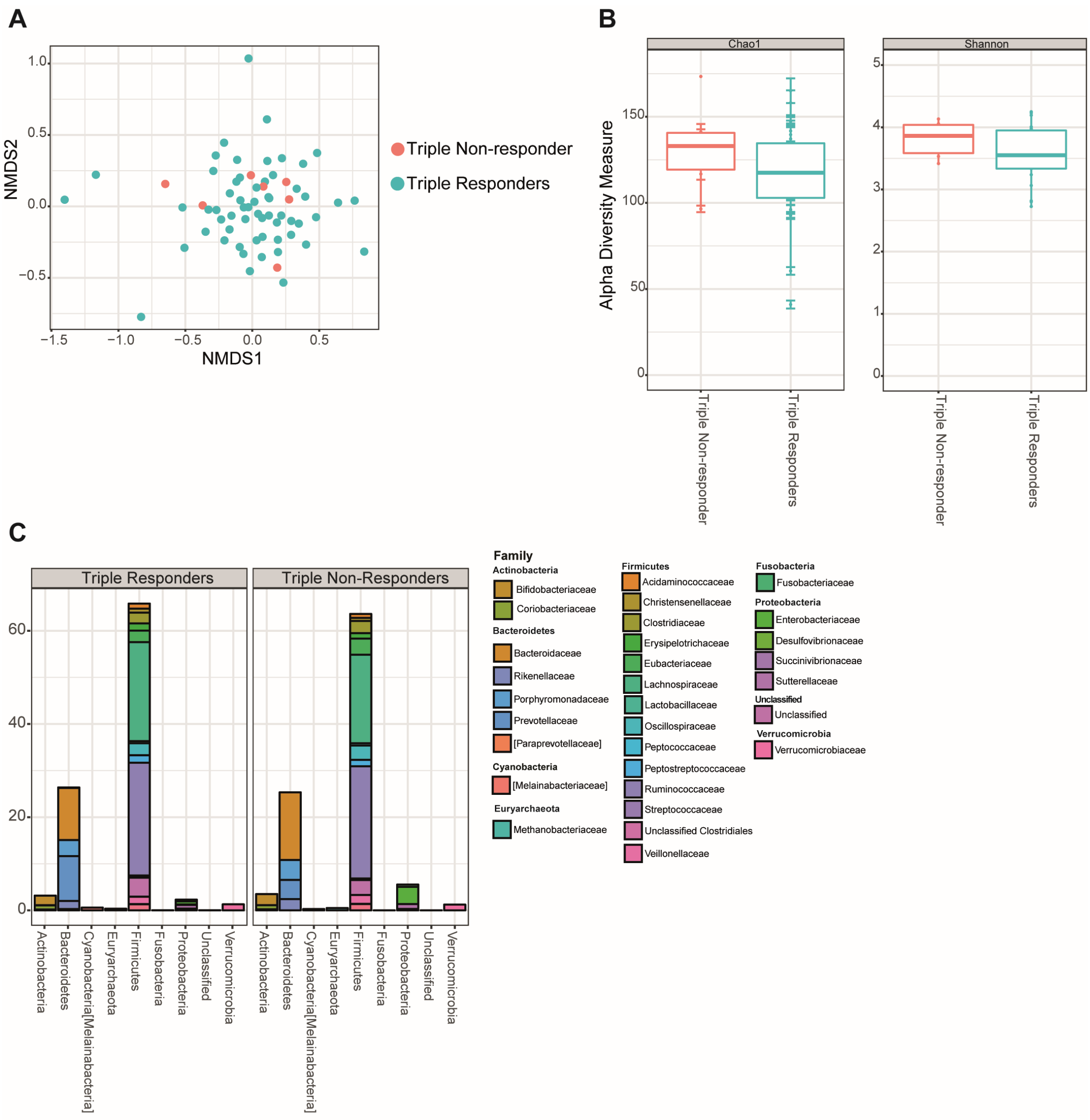

Figure S8: Microbiome analysis. Microbiota composition analysis using 16S rRNA sequencing of triple responders and triple non-responders. (A) β-diversity analysis of fecal microbiota using NMDS. (B) Quantification of α-diversity using Chao1 and Shannon index plots comparing triple responders and triple non-responders. (C) Relative abundances of bacterial families are shown grouped by phylum.

Supplementary Table 1

Study	Vaccinee	Gender	Age	Infection Incidence URTIs (last 12 months)	Infection Incidence LRTIs (last 12 months)	Asthma	Rheuma	Previous Influenza Vaccinations	Frequency of Influenza Vaccinations	Last Influenza Vaccination	Medication (Rheuma, Immunesuppressives)	Medication (Antibiotics)
1st Study	Responders	female	68	0	0	no	no	yes	less than almost every year	-	-	-
		female	67	1	0	no	no	no	-	-	no	no
		female	68	1	0	no	yes	yes	less than almost every year	2008	yes	no
		female	70	0	0	no	no	yes	less than almost every year	-	no	no
		male	71	2	0	no	yes	yes	less than almost every year	2011	no	no
		female	66	3	0	no	no	yes	almost every year	2013	no	no
		female	67	0	0	no	no	no	less than almost every year	-	no	no
	Non-Responders	male	65	2	0	no	no	yes	only once	2012	no	no
		male	70	0	0	no	no	yes	every year	2013	no	no
		male	69	0	0	no	no	yes	almost every year	2013	no	no
		female	79	1	0	no	no	yes	every year	2013	no	no
		female	70	2	2	yes	no	yes	every year	2013	no	no
		female	71	3	0	no	no	yes	every year	2013	no	no
2nd Study	Responders	male	76	2	0	no	no	no	-	-	no	no
		female	66	2	0	no	no	yes	less than almost every year	2012	no	no
		male	66	0	0	no	yes	no	-	-	no	no
		female	77	1	1	no	no	no	-	-	-	-
		male	71	2	0	no	no	yes	only once	2010	no	no
		male	65	0	0	no	-	yes	less than almost every year	-	yes	-
		male	72	4	0	no	no	no	-	-	no	no
		male	67	3	0	no	no	yes	only once	2005	no	no
		male	74	2	0	no	no	no	-	-	no	no
		female	72	0	0	no	no	no	-	-	no	no
	Non-Responders	male	72	0	0	no	no	yes	every year	2014	no	no
		female	67	1	0	no	yes	yes	every year	2014	yes	no
		female	74	1	1	no	no	yes	every year	2014	no	no
		male	76	1	0	no	-	yes	only once	-	no	no
		male	75	2	0	no	no	yes	almost every year	2013	no	no
		male	74	1	1	no	no	yes	every year	2014	no	no
		male	67	0	0	no	yes	yes	only once	2010	yes	-
		male	72	1	0	no	no	yes	every year	2014	no	no
		male	80	2	0	no	no	yes	every year	2014	no	no
		male	74	2	0	no	no	yes	every year	2014	no	no

URTI = Upper Respiratory Tract Infection
LRTI = Lower Respiratory Tract Infection

Supplementary Table 1: Background information on the assessed triple vaccine responders and non-responders and the available datasets. The shown data originates from questionnaires filled in by the study participants. The answers for the infection incidence are coded as follows: 0 = no infection, 1 = once, 2 = twice, 3 = 3 to 4 times, 4 = more than 4 times.

Supplementary Table 2

Method/ Analysis	Study	Data Available	n
HAI Assay	1st	yes	34
	2nd	yes	200
MN Assay	1st	yes	13°
	2nd	yes	20°
FC - Multifunctional CD4+ & CD8+ T Cells	1st	yes	12
	2nd	yes	18#
FC - T_{REG} & T_{FH} Cells	1st	yes	12
	2nd	yes	13#
FC - B Cell Populations	1st	yes	12
	2nd	yes	13#
FC - Memory T Cell Populations	1st	yes	12
	2nd	yes	18#
Procartaplex Analysis	1st	yes	13
	2nd	yes	20
Pathway Annotation (Pathview)	1st	no	-
	2nd	yes	20
Bulk Transcriptome Analysis	1st	yes, not shown§	-
	2nd	yes	20
scRNA Sequencing	1st	no	-
	2nd	yes	6
Mathematical Modeling	1st	yes, not shown*	-
	2nd	yes	(according to FC Data)

FC = Flow Cytometry
* Data is not shown due to very small number of input data and consequent poor robustness.
° Data is available for n=234 in total but only shown for the indicated subgroups.
# Exclusion of samples/data not available due to low cell numbers.
§ Data not shown/included due to poor quality.

Supplementary Table 2: Background information on data availability and sample size.

Supplementary Table 3

GMTs before vaccination (day 0) and 21/70 days post vaccination determined by HAI assay:

		1st study			2nd study		
		**Pre-vaccination (day 0)**	**Post-vaccination (day 21/70)**	***p*-value***	**Pre-vaccination (day 0)**	**Post-Vaccination (day 21/70)**	***p*-value***
**GMT (±SD)**							
A/H1N1	Responders	1.9 (±27.6)	1159.3 (±799.7)	0.0156	1.3 (±2.7)	452.5 (±1437)	0.0020
	Non-Responders	179.6 (±89.4)	77.1 (±116.8)	0.7500	37.3 (±93.4)	49.2 (±116.3)	0.1250
A/H3N2	Responders	61.6 (±206.6)	1902.1 (±1747.9)	0.0156	4.3 (±95.7)	2743.7 (±7600,7)	0.0020
	Non-Responders	142.5 (±208.8)	113.1 (±40)	0.6250	31.1 (±67.9)	43.9 (±112.9)	0.0625
B	Responders	48.8 (±45.3)	353.3 (±207)	0.0156	1.7 (±6.0)	259.9 (±323.6)	0.0020
	Non-Responders	50.4 (±24.3)	71.3 (±53.7)	0.2500	13.5 (±44.8)	12.6 (±45.2)	> 0.9999

* Significance comparison between pre- and post-vaccination GMT within a group (Wilcoxon test (paired, non-parametric, two-tailed)).

GMTs before vaccination (day 0) and 21/70 days post vaccination determined by MN assay:

		1st study			2nd study		
		**Pre-vaccination (day 0)**	**Post-vaccination (day 21/70)**	***p*-value***	**Pre-vaccination (day 0)**	**Post-Vaccination (day 21/70)**	***p*-value***
**GMT (±SD)**							
A/H1N1	Responders	1 (±0)	70.2 (±218.1)	0.0313	1.4 (±11.7)	167.7 (±1482)	0.0039
	Non-Responders	10.4 (±55.7)	14.7 (±53.5)	0.2500	4.1 (±24.6)	8.5 (±48.1)	0.2500
A/H3N2	Responders	6.5 (±39.1)	579.7 (±471.8)	0.0156	11.5 (±417.8)	849.6 (±16472)	0.0059
	Non-Responders	24.3 (±29)	63.5 (±43.8)	0.0625	26.7 (±58.9)	100.9 (±276.1)	0.0078
B	Responders	4.4 (±17.1)	262.5 (±375.5)	0.0156	3.2 (±94.7)	452.5 (±1425)	0.0020
	Non-Responders	46.6 (±46.3)	38.5 (±48.3)	0.5625	22.5 (±396.2)	179.7 (±793.1)	0.0078

* Significance comparison between pre- and post-vaccination GMT within a group (Wilcoxon test (paired, non-parametric, two-tailed)).

Supplementary Table 3: Summary of HAI and MN titres before and 21/70 days post vaccination for all single antigens contained in the vaccine formulation in both respective seasons. Shown is the geometric mean titre (GMT) ± SD.

Supplementary Methods

Microbial 16S rRNA gene analysis:
Fecal pellets were collected, immediately frozen and maintained at -20°C until processing. DNA Isolation was done using a phenol-chloroform based protocol (Turnbaugh et al., 2009). The methods used for 16S rRNA sequencing analyses are based on the Human Microbiome Project HMP (Human Microbiome Project Consortium, 2012). In brief, we employed Illumina MiSeq 250 bp paired-end sequencing of the hypervariable V4 region. Obtained reads were assembled, quality controlled and clustered using the QIIME v1.8.0 (Quantitative Insights into Microbial Ecology) analysis pipeline (Caporaso et al., 2010). In short, quality filtering was set up to -q 30, minimum read length 200 bp and minimum number of sequences per sample = 1000. The OTU clusters and representative sequences were determined using open-reference OTU picking using UCLUST at 97% identity (Edgar, 2010), followed by abundance filtering (OTUs cluster > 0.5%; or as indicated otherwise) and taxonomy assignment using the RDP Classifier (Wang et al., 2007) with a bootstrap confidence cutoff of 80%. The OTU absolute abundance table and mapping file are used for statistical analyses and data visualization in the R statistical programming environment (R Development Core Team, 2011), package PHYLOSEQ (McMurdie and Holmes, 2013)

Caporaso, J.G., Kuczynski, J., Stombaugh, J., Bittinger, K., Bushman, F.D., Costello, E.K., Fierer, N., Peña, A.G., Goodrich, J.K., Gordon, J.I., et al. (2010). QIIME allows analysis of high-throughput community sequencing data. Nat. Methods 7, 335–336.

Edgar, R.C. (2010). Search and clustering orders of magnitude faster than BLAST. Bioinformatics 26, 2460–2461.

Human Microbiome Project Consortium (2012). A framework for human microbiome research. Nature 486, 215–221.

McMurdie, P.J., and Holmes, S. (2013). phyloseq: an R package for reproducible interactive analysis and graphics of microbiome census data. PLoS One 8, e61217.

R Development Core Team (2011). R: A language and environment for statistical computing R Foundation for Statistical Computing (Vienna, Austria : the R Foundation for Statistical Computing: ISBN: 3-900051-07-0. Available online at http://www.R-project.org/.).

Turnbaugh, P.J., Hamady, M., Yatsunenko, T., Cantarel, B.L., Duncan, A., Ley, R.E., Sogin, M.L., Jones, W.J., Roe, B.A., Affourtit, J.P., et al. (2009). A core gut microbiome in obese and lean twins. Nature 457, 480–484.

Wang, Q., Garrity, G.M., Tiedje, J.M., and Cole, J.R. (2007). Naive Bayesian classifier for rapid assignment of rRNA sequences into the new bacterial taxonomy. Appl. Environ. Microbiol. 73, 5261–5267.
