# Supplementary material for: Distinct immunological and molecular signatures underpinning influenza vaccine responsiveness in the elderly
Source: Nat Commun. 2022 Nov 12;13:6894. doi: 10.1038/s41467-022-34487-z (PMC9653450; doi:10.1038/s41467-022-34487-z)
Supplement: Supplementary file 2 — Reporting Summary [file 41467_2022_34487_MOESM2_ESM.pdf]

nature portfolio

Corresponding author(s): Peggy Riese

Last updated by author(s): 2022/10/05

Reporting Summary

Nature Portfolio wishes to improve the reproducibility of the work that we publish. This form provides structure for consistency and transparency in reporting. For further information on Nature Portfolio policies, see our Editorial Policies and the Editorial Policy Checklist.

Statistics

For all statistical analyses, confirm that the following items are present in the figure legend, table legend, main text, or Methods section.

n/a	Confirmed	
☐	☒	The exact sample size (*n*) for each experimental group/condition, given as a discrete number and unit of measurement
☐	☒	A statement on whether measurements were taken from distinct samples or whether the same sample was measured repeatedly
☐	☒	The statistical test(s) used AND whether they are one- or two-sided<br>*Only common tests should be described solely by name; describe more complex techniques in the Methods section.*
☒	☐	A description of all covariates tested
☐	☒	A description of any assumptions or corrections, such as tests of normality and adjustment for multiple comparisons
☐	☒	A full description of the statistical parameters including central tendency (e.g. means) or other basic estimates (e.g. regression coefficient) AND variation (e.g. standard deviation) or associated estimates of uncertainty (e.g. confidence intervals)
☐	☒	For null hypothesis testing, the test statistic (e.g. *F*, *t*, *r*) with confidence intervals, effect sizes, degrees of freedom and *P* value noted<br>*Give P values as exact values whenever suitable.*
☒	☐	For Bayesian analysis, information on the choice of priors and Markov chain Monte Carlo settings
☒	☐	For hierarchical and complex designs, identification of the appropriate level for tests and full reporting of outcomes
☒	☐	Estimates of effect sizes (e.g. Cohen's *d*, Pearson's *r*), indicating how they were calculated

Our web collection on statistics for biologists contains articles on many of the points above.

Software and code

Policy information about availability of computer code

Data collection	Luminex® 100/200™ System (provided software), BD FACSDiva™ Software
Data analysis	GraphPad Prism (version 6.0 and 8.4.3 for Windows, GraphPad Software, La Jolla California USA, www.graphpad.com), FlowJo_v10.8.0, Cell Ranger 3.0.1 (http://10xgenomics.com), R/Seurat package v3.1, MATLAB 2016a, Pathview Online Tool

For manuscripts utilizing custom algorithms or software that are central to the research but not yet described in published literature, software must be made available to editors and reviewers. We strongly encourage code deposition in a community repository (e.g. GitHub). See the Nature Portfolio guidelines for submitting code & software for further information.

Data

Policy information about availability of data

All manuscripts must include a data availability statement. This statement should provide the following information, where applicable:

- Accession codes, unique identifiers, or web links for publicly available datasets
- A description of any restrictions on data availability
- For clinical datasets or third party data, please ensure that the statement adheres to our policy

Source data are provided with this paper. Bulk genome and scRNA sequencing data generated in this study have been deposited in the data base https://www.ncbi.nlm.nih.gov/geo/ under the accession codes GSE211368 and GSE211560, respectively. Microbiome sequencing data of all samples used in this study was deposited to a public repository with the Accession no./ Bioproject: PRJEB56539.

Field-specific reporting

Please select the one below that is the best fit for your research. If you are not sure, read the appropriate sections before making your selection.

☒ Life sciences ☐ Behavioural & social sciences ☐ Ecological, evolutionary & environmental sciences

For a reference copy of the document with all sections, see nature.com/documents/nr-reporting-summary-flat.pdf

Life sciences study design

All studies must disclose on these points even when the disclosure is negative.

Sample size	A review paper of Goodwin et al. reported seroconversion rates for influenza vaccination among elderly individuals in 31 studies from 12 high-income countries. The pooled estimate for H1N1 was 42%, for H3N2 51% and for B type 35%. In a sample of 200 elderly individuals we thus expected around 115, 100 and 130 nonresponders to H1N1, H3N2 and B viruses, respectively. For instance, in a model with 100 nonresponders, one will be able to simultaneously analyze at least 10 predictors of poor responsiveness to influenza vaccination.
Data exclusions	For the flow cytometry data, single samples were excluded if a preparation/measurement effect was obvious that rendered the yielded information unuseful.
Replication	The findings from the 1st study were tested by the findings from the 2nd study. The 2nd study supported the findings of the 1st, smaller study.
Randomization	Donors/samples were allocated to the experimental groups according to their HAI titre (seroconversion post vaccination).
Blinding	The investigators performed the stratification of the vaccinees in order to identify samples for the further analysis. Therefore, blinding to group allocations was not possible for the further analysis

Reporting for specific materials, systems and methods

We require information from authors about some types of materials, experimental systems and methods used in many studies. Here, indicate whether each material, system or method listed is relevant to your study. If you are not sure if a list item applies to your research, read the appropriate section before selecting a response.

Materials & experimental systems

n/a	Involved in the study	
☐	☒	Antibodies
☒	☐	Eukaryotic cell lines
☒	☐	Palaeontology and archaeology
☒	☐	Animals and other organisms
☐	☒	Human research participants
☒	☐	Clinical data
☒	☐	Dual use research of concern

Methods

n/a	Involved in the study	
☒	☐	ChIP-seq
☐	☒	Flow cytometry
☒	☐	MRI-based neuroimaging

Antibodies

Antibodies used	CD4 (PE-Cy7, L200, Cat.-No. 560909, dilution 1:200), CD19 (APC-Cy7, SJ25C1, Cat.-No. 560177, dilution 1:100), CD14 (APC-Cy7, MΦP9, Cat.-No. 560180, dilution 1:100), CD45RA (FITC, HI100, Cat.-No. 555488, dilution 1:100) and CD57 (PE, NK-1, Cat.-No. 560844, dilution 1:100) from BD (New Jersey, USA); CD4 (BV510, OKT4, Cat.-No. 317444, dilution 1:100), CD8 (BV650, RPA-T8/A700, SK1, Cat.-No. 344730, dilution 1:200), CCR7 (BV785, G043H7, Cat.-No. 353230, dilution 1:100), CD28 (PE-Cy5, CD28.2, Cat.-No. 302910, dilution 1:100), CD24 (BV605, ML5,Cat.-No. 311124, dilution 1:50), IFNγ (A700, 4S.B3, Cat.-No. 502520, dilution 1:200), TNFα (BV605, Mab11, Cat.-No. 502936, dilution 1:200), IL-2 (PB, MQ1-17H12, Cat.-No. 551383, dilution 1:100), IL-10 (PE-Dazzle594, JES3-19F1, Cat.-No. 506812, dilution 1:200), CD127 (BV605, A7R34A019D5, Cat.-No. 351334, dilution 1:50 / BV711, A019D5, Cat.-No. 351328, dilution 1:200), CD25 (BV650, BC96, Cat.-No. 302634, dilution 1:50), ICOS(BV421, C398.4A, Cat.-No. 313524, dilution 1:100), CXCR5 (PE-Cy7, J252D4, Cat.-No. 356924, dilution 1:300) and FOXP3 (FITC, 206D, Cat.-No. 320112, dilution 1:100) from BioLegend (San Diego, USA); FOXP3 (APCAF647, 236A/E7206D, Cat.-No. 320113, dilution 1:100) from eBioscience/ ThermoFisher Scientific (Massachusetts, USA); and CD3 (BUV395, SK7, Cat.-No. 564001, dilution 1:200), CD19 (A700, HIB19, Cat.-No. 557921, dilution 1:200), CD27 (PerCP-Cy5.5, M-T271, Cat.-No. 560612, dilution 1:100), CD38 (PE-Cy7, HB7, Cat.-No. 335825, dilution 1:100) and CD20 (PE-Cy5, 2H7, Cat.-No. 555624, dilution 1:100) from BD (Franklin Lakes, New Jersey, USA). CD3 (AF 488 (, UCHT1, Cat.-No.300415, dilution 1:200), CD4 (AF 647, (RPA-T4, Cat.-No. 300520, dilution 1:200) conjugates (BioLegend).
Validation	All used antibodies are validated/ quality control tested for the analysis of human cells by flow cytometry according to the manufacturer's information.

Human research participants

Policy information about studies involving human research participants

Population characteristics	The only relevant population characteristic defining recruitment was the age. Participants had to be aged 65 or older. The recruited participants were 65-80-year-old. Exclusion criteria were an allery to egg protein, an acute infection, restricted cognitive skills and a prior vaccination against influenza in the respective season. Female and male participants were included in the study. Information on past and current diagnosis and treatment categories include infection incidence of URTIs and LRTIs, Asthma, Rheuma, influenza vaccinations as well as antibiotoc and immunesuppressive medication.
Recruitment	Participants were randomly recruited from the general population of Hannover, Germany (letters were sent to ndividuals randomly selected from the residents' registration office, n=600 1st study, n=6000 2nd study). The only restriction/bias was the age of the participants. The age and sex distribution was chosen representative of the general German population in this age group.The representativeness of these random population-based samples was verified by a non-participant survey (Akmatov et al., BMC Med Res Methodol 2017). We oversampled the older age group (76–80 years) to ensure an adequate sample size in this age group.
Ethics oversight	The study was approved by the ethics committee of the Hannover Medical School and participants gave written informed consent to the use of their data.

Note that full information on the approval of the study protocol must also be provided in the manuscript.

Flow Cytometry

Plots

Confirm that:

☒ The axis labels state the marker and fluorochrome used (e.g. CD4-FITC).

☒ The axis scales are clearly visible. Include numbers along axes only for bottom left plot of group (a 'group' is an analysis of identical markers).

☒ All plots are contour plots with outliers or pseudocolor plots.

☒ A numerical value for number of cells or percentage (with statistics) is provided.

Methodology

Sample preparation	For surface staining cells were incubated with the antibody mix prepared in PBS for 30 min at 4°C in the dark. For intracellular and intranuclear staining, cells were permeabilized using the Cytofix/Cytoperm kit (BD) or the FOXP3 staining kit (eBioscience), respectively, according to the manufacturer’s instructions. Antibodies were diluted in the respective wash buffer.
Instrument	BD Fortessa flow cytometer
Software	Diva software for acquisition and FlowJo for analysis
Cell population abundance	For the sorting of CD4+ T cells, PBMCs were labelled with anti-CD3-AF 488 (UCHT1) and anti-CD4-AF 647 (RPA-T4) conjugates (BioLegend). Dead cells were excluded using the LIVE/DEAD™ Fixable Near-IR Dead Cell Stain Kit (ThermoFisher Scientific, Waltham, USA). Cells were sorted as living CD3+CD4+ cells.
Gating strategy	For the identification of the indicated cell populations, a time gate (SSC-W vs. time) was used to exclude any measurement inconsistencies. Living cells were selected by plotting FSC-A vs. Life/Dead staining. Subsequently, single lymphocytes (FSC-A vs. SSC-A, doublet exclusion by FSC-A vs. FSC-H and SSC-A vs. SSC-W) were selected. For the characterization of T cell subsets, CD3-expressing cells (CD8 vs. CD3) were split into CD4+ and CD8+ T cells (CD4 vs. CD8). For the analysis of cytokines or single analytes, these cell populations were plotted against the respective marker. For the identification of memory populations, CCR7- and CCR7+ populations were identified by plotting CD4 or CD8 against CCR7. For the subsequent detection of CD45RA + and CD45RA- populations, a histogram was used. For the identification of T helper subsets dependent on surface marker expression CD4+CCR7+CD45RA+ naïve T cells were plotted against CXCR3 und positive and negative populations were plotted against CCR6. For the identification of Tfh and Treg cells, CD4+ T cells were selected (CD4 vs. CD3) and CXCR5+ICOS+ cells were selected as Tfh cells. CD4+CD127lowCD25+FOXP3+ cells were selected as Treg cells. For the B cell analysis, B cells (CD19 +CD3-) were selected and plotted for the expression of CD27 (memory B cells), CD24 vs. CD38 (CD24hiCD38hi = transitional B cells) and memory B cells were further plotted CD20 vs. CD38 for the identification of plasmablasts (CD20-CD38+). The identification of all cell populations was performed using unstained and single stained control samples as well as maximal stimulation controls (SEB).<br>A comprehensive gating strategy will be included in the final manuscript version.

☐ Tick this box to confirm that a figure exemplifying the gating strategy is provided in the Supplementary Information.
